# Methyltransferase-Like 3 Inhibition-Activated cGAS/STING Axis Enhances Immunotherapy and Poly(ADP-Ribose) Polymerase Inhibitor Sensitivity in Lung Adenocarcinoma

**DOI:** 10.34133/research.1265

**Published:** 2026-05-20

**Authors:** Jiawang Zhou, Jiaxin He, Yunqing Lu, Cheng Yi, Xing Chang, Lijun Tao, Ke Zhong, Haisheng Zhang, Hongxin Niu, Xiansong Wang, Zhuojia Chen, Hongsheng Wang

**Affiliations:** ^1^ Guangdong Provincial Key Laboratory of Chiral Molecule and Drug Discovery, State Key Laboratory of Anti-Infective Drug Discovery and Development, School of Pharmaceutical Sciences, Sun Yat-sen University, Guangzhou 510006, China.; ^2^ Department of Physiology, School of Basic Medical Sciences, Gannan Medical University, Ganzhou 341000, China.; ^3^ Department of General Practice, Zhujiang Hospital, Southern Medical University, Guangzhou 510282, China.; ^4^ Special Medical Service Center, Zhujiang Hospital, Southern Medical University, Guangzhou 510282, China.; ^5^ Sun Yat-sen University Cancer Center, State Key Laboratory of Oncology in South China, Collaborative Innovation Center for Cancer Medicine, Guangzhou 510060, China.

## Abstract

The cyclic GMP-AMP synthase (cGAS)/stimulator of interferon genes (STING)-mediated type I interferon response plays a crucial role in antitumor immunity, yet the regulatory factors involved in this innate immune response remain incompletely understood. Here, we identified methyltransferase-like 3 (METTL3), an RNA *N^6^*-methyladenosine methyltransferase, as a key suppressor of the cGAS/STING pathway in lung adenocarcinoma (LUAD) cells. METTL3 was overexpressed in LUAD tissues, and elevated METTL3 expression was associated with decreased CD8^+^ T-cell infiltration and poor clinical outcomes. Mechanistically, METTL3 deficiency promoted nuclear DNA leakage into the cytoplasm, thereby activating the cGAS pathway and enhancing antitumor immunity. Specifically, METTL3 knockdown decreased the efficiency of homologous recombination repair by down-regulating MutS homolog 5, leading to cytosolic DNA accumulation. On the other hand, *N^6^*-methyladenosine methylation of *cGAS* messenger RNA destabilized the transcript and suppressed its protein expression. Functionally, METTL3 knockdown sensitized LUAD cells to poly(ADP-ribose) polymerase inhibitors. In vivo and clinical data confirmed the beneficial effects of METTL3-inhibition-induced activation of the cGAS/STING axis on tumor growth and LUAD progression. Collectively, our findings reveal that METTL3 inhibition activates cGAS/STING-mediated antitumor immunity by inducing cytosolic DNA accumulation and cGAS up-regulation, thereby modulating poly(ADP-ribose) polymerase inhibitor response and cancer progression in LUAD.

## Introduction

Lung adenocarcinoma (LUAD), the most prevalent histological subtype of non-small-cell lung cancer, accounts for approximately 40% of all lung cancer cases. Globally, it ranks among the leading causes of cancer-related morbidity and mortality, posing a notable public health concern [1]. In recent years, immunotherapy has emerged as a groundbreaking modality for LUAD treatment, leading to major progress in cancer care [2,3]. However, the heterogeneous pathologies, drug resistance, and recurrence of LUAD present formidable challenges for effective treatment. Among these obstacles, immunosuppression and resistance to immunotherapy are particularly prominent [4]. These limitations highlight the need to integrate immunotherapy into a comprehensive treatment approach, leveraging combined effects to improve clinical efficacy and target tumors more effectively, which represents the current frontier of cancer therapy research [5]. Consequently, developing innovative strategies that synergistically augment immunotherapy for LUAD has become a critical and urgent priority for scientific investigation.

*N^6^*-methyladenosine (m^6^A) is the most abundant internal chemical modification in eukaryotic messenger RNAs (mRNAs) [6–8]. Accumulating evidence suggests that m^6^A, along with its regulatory enzymes and reader proteins, plays critical roles in posttranscriptional gene regulation, and that the dysregulation of these genes is associated with various diseases, especially cancer [9–11]. Methyltransferase-like 3 (METTL3), a well-characterized m^6^A writer, serves as a key component of the methyltransferase complex and is crucial for establishing m^6^A modification [12]. Notably, METTL3 has been implicated in immunotherapy responses in multiple cancers [13,14], and its inhibition has been shown to reprogram the tumor microenvironment and enhance the efficacy of anti-PD-1 therapy [15]. A deeper understanding of the role and the underlying molecular mechanisms of METTL3 in anticancer immunity will accelerate the development of effective METTL3-targeted therapeutics, offering promising avenues for improving cancer immunotherapy.

DNA damage induces the accumulation of cytosolic DNA fragments within cells [16], which stimulate type I interferon (IFN) production through the cyclic GMP-AMP synthase (cGAS)/stimulator of interferon genes (STING) signaling pathway. Specifically, cytosolic DNA binds to cGAS, which then catalyzes the production of the second messenger cyclic guanosine monophosphate (cGAMP)[17]. Subsequently, cGAMP interacts with and activates STING, leading to the recruitment and activation of TANK-binding kinase 1, initiating the interferon regulatory factor 3 and nuclear factor kappa-B signaling cascades and cytokine production [18]. Therapeutic reactivation of the cGAS/STING signaling pathway has emerged as a promising strategy for cancer treatment. For instance, combining STING agonists with carboplatin has been shown to reduce tumor burden and prolong survival in mouse models [19]. Thus, activating the cGAS/STING pathway represents a pivotal strategy for potentiating tumor immunotherapy.

The posttranslational regulation of cGAS/STING signaling has been extensively studied [20]. Previous studies have demonstrated that TRIM14 recruits USP14 to cleave the lysine 48 (K48)-linked ubiquitin chain of cGAS at K414, thereby inhibiting p62-mediated autophagic degradation of cGAS and increasing type I interferon signal activation [21]. USP35 can directly deubiquitinate and inactivate STING through phosphorylation of STING at Ser366 [22]. However, evidence connecting the cGAS/STING signaling pathway and epigenetic regulatory factors is limited.

Although posttranscriptional regulators are potent and promising targets for tumor immunotherapy, whether RNA modifications, such as m^6^A, directly regulate the cGAS/STING pathway and affect antitumor immunity remains unknown. In the present study, we investigated the potential effects of m^6^A on the activation of cGAS-mediated antitumor immunity in LUAD cells. Our data reveal that METTL3 inhibition activates cGAS-mediated antitumor immunity via the accumulation of cytosolic DNA and up-regulation of cGAS expression, thereby regulating poly(ADP-ribose) polymerase (PARP) inhibitor response and cancer progression in LUAD.

## Results

### METTL3 expression is correlated with immune cell infiltration and cancer progression

**Fig. 1.**
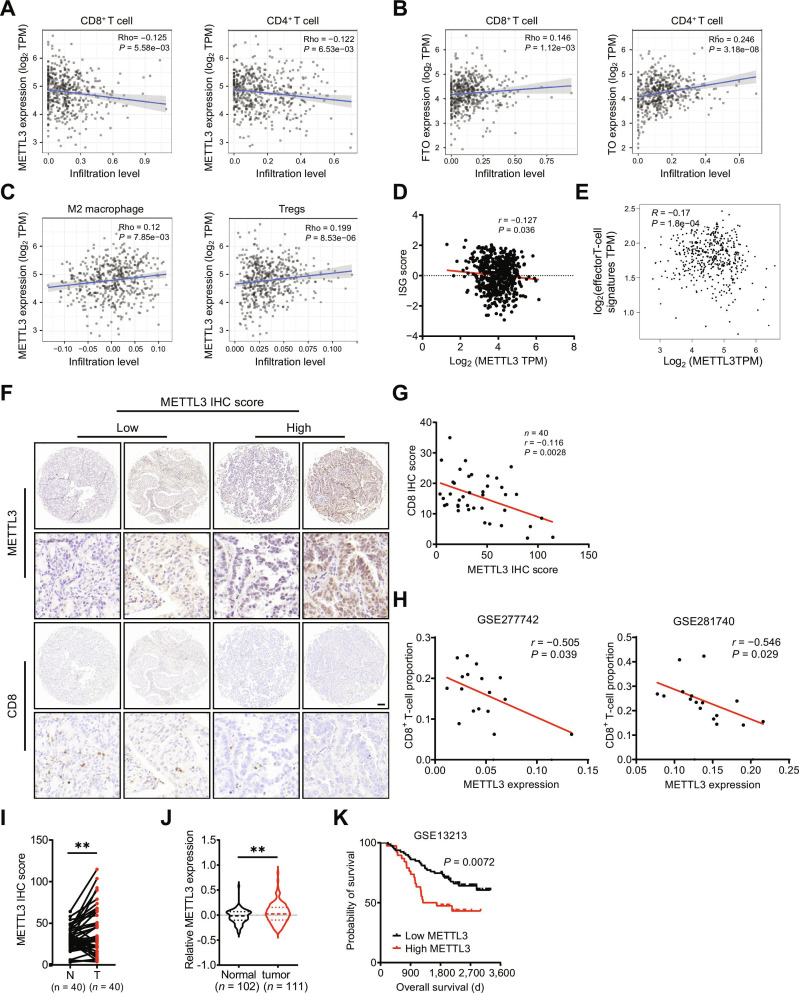
Methyltransferase-like 3 (METTL3) expression is correlated with immune cell infiltration and cancer progression. (A) The correlation between the expression of METTL3 and the infiltrating level of CD8^+^ T cells or CD4^+^ T cells in lung adenocarcinoma (LUAD) based on the TIMER platform. TPM, transcripts per million. (B) The correlation between the expression of fat mass and obesity-associated protein (FTO) and the infiltrating level of CD8^+^ T cells or CD4^+^ T cells in LUAD based on the TIMER platform. (C) The correlation between the expression of METTL3 and the infiltrating level of M2 macrophages or regulatory T cells (Tregs) in LUAD based on the TIMER platform. (D) The correlation between the expression of METTL3 and ISG scores in LUAD based on the TCGA database. (E) The correlation between the expression of METTL3 and effector T-cell markers (including CX3CR1, FGFBP2, and FCGR3A) in LUAD based on the TCGA database. (F) Representative IHC staining images of CD8 in LUAD tissues with high or low immunohistochemical (IHC) scores of METTL3; scale bar = 100 μm. (G) The correlation between the IHC scores of METTL3 and the IHC scores of CD8 in LUAD tissues. (H) The correlation between the expression of METTL3 and infiltrating level of CD8^+^ T cells in GSE277742 and GSE281740. (I) The IHC scores of METTL3 in LUAD tumor tissues and adjacent normal mucosa tissues. (J) The expression of METTL3 in LUAD tumor tissues and adjacent normal mucosa tissues from the CPTAC database. (K) Kaplan–Meier curve analysis of overall survival in the GSE13213 database by the expression of METTL3. Data are presented as mean ± SD from 3 independent experiments. **P < 0.01, by Student’s *t* test between 2 groups and by one-way analysis of variance (ANOVA) followed by Bonferroni test for multiple comparison.

To elucidate the potential pathological roles of METTL3 in the antitumor immune response within LUAD, we initially investigated the correlation between METTL3 expression and immune cell infiltration using the TIMER2.0 web server (http://timer.comp-genomics.org/) [23]. The results revealed that METTL3 expression was negatively correlated with the infiltration of various antitumor immune cells, including CD8^+^ T cells (*r* = −0.125, *P* = 5.58e−03), CD4^+^ T cells (*r* = −0.122, *P* = 6.53e−03), M1 macrophages (*r* = −0.139, *P* = 2.01e−03), monocytes (*r* = −0.123, *P* = 6.30e−03), and neutrophils (*r* = −0.172, *P* = 1.24e−03) (Fig. 1A and Fig. S1A). Conversely, the expression of the m^6^A demethylase fat mass and obesity-associated protein (FTO) was positively correlated with the infiltration of these antitumor immune cells (CD8^+^ T cells, *r* = 0.146, *P* = 1.12e−03; CD4^+^ T cells, *r* = 0.246, *P* = 3.18e−08) (Fig. 1B). Furthermore, METTL3 expression was positively correlated with the infiltration of diverse protumor immune cells, including M2 macrophages (*r* = 0.12, *P* = 7.85e−03) and regulatory T cells in LUAD tissues (*r* = 0.199, *P* = 8.53e−03) (Fig. 1C). Notably, similar correlations were observed in several other types of cancers (Fig. S1B), suggesting that METTL3 is broadly involved in modulating immune-cell infiltration within tumor microenvironments.

Using a previously established method for calculating the interferon-stimulated gene (ISG) score based on the expression profile of a 38-gene signature [24], we observed that elevated METTL3 expression in LUAD was associated with lower ISG scores (Fig. 1D). Additionally, the expression of effector T-cell markers (including *CX3CR1*, *FGFBP2*, and *FCGR3A*) [25], which mediate immune effector functions, was negatively correlated with METTL3 expression (*r* = −0.17, *P* = 1.8e−04) (Fig. 1E) but positively correlated with FTO expression (*r* = 0.29, *P* = 1.7e−10) (Fig. S1C) in LUAD. Furthermore, immunohistochemical (IHC) staining revealed markedly reduced intratumoral CD8^+^ T-cell infiltration in LUAD samples with high METTL3 expression (Fig. 1F and Fig. S1D), and METTL3 protein levels were negatively correlated with CD8^+^ cell infiltration (Fig. 1G). Moreover, analysis of 2 independent single-cell RNA sequencing datasets (GSE277742 and GSE281740 [26]) consistently demonstrated a significant negative correlation between METTL3 expression and CD8^+^ T-cell infiltration (*r* = −0.505, *P* = 3.9e−02; *r* = −0.546, *P* = 2.9e−02, respectively) (Fig. 1H). Collectively, these findings indicate that METTL3 plays a critical role in immune regulation within LUAD.

Beyond its up-regulation in LUAD tissues, METTL3 expression was found to be elevated in several other cancer types, including colon adenocarcinoma, esophageal carcinoma, and prostate adenocarcinoma, compared to corresponding normal adjacent tissues (Fig. S1E). Consistent with this, METTL3 protein levels were significantly higher in LUAD patients as demonstrated by IHC staining (Fig. 1I) and the Clinical Proteomic Tumor Analysis Consortium (CPTAC) database (Fig. 1J). Moreover, elevated METTL3 expression was associated with poorer overall survival in LUAD patients (Fig. 1K). Taken together, these findings support an inverse relationship between METTL3 expression and the infiltration of antitumor immune cells while highlighting the oncogenic role of METTL3 in LUAD progression.

### METTL3 knockdown promotes the innate immune response and activates the cGAS–STING pathway

**Fig. 2.**
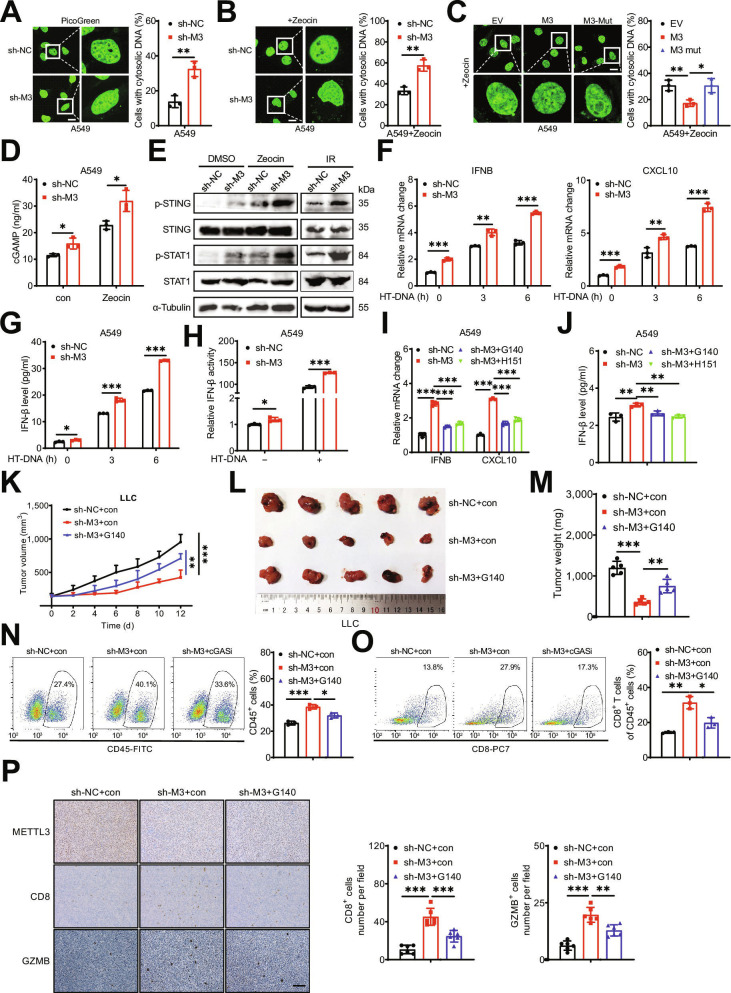
Knockdown of METTL3 promotes the innate immune response and activates the cyclic GMP-AMP synthase (cGAS)–stimulator of interferon genes (STING) pathway. (A) Representative confocal images of PicoGreen stain in sh-control and sh-METTL3 A549 cells (left), and the percentages of cells displaying cytosolic DNA were measured (right); scale bar = 20 μm. (B) Representative confocal images of PicoGreen stain in sh-control and sh-METTL3 A549 cells treated with Zeocin (left), and the percentages of cells displaying cytosolic DNA were measured (right); scale bar = 20 μm. (C) Representative confocal images of PicoGreen stain in A549 cells with Zeocin treatment, transfected with vector control, METTL3 wild-type (WT) plasmid, and METTL3 DA mutant plasmid for 24 h (left), and the percentages of cells displaying cytosolic DNA were measured (right), scale bar = 20 μm; (D) Intracellular cGAMP levels in sh-control and sh-METTL3 A549 cells with and without Zeocin treatment. (E) The protein expression of phosphorylation of STING (p-STING) and STAT1 (p-STAT1) in sh-control and sh-METTL3 A549 cells with or without Zeocin, and ionizing radiation (IR) treatment. (F) The messenger RNA (mRNA) levels of IFNB1 and CXCL0 mRNA in sh-control and sh-METTL3 A549 cells treated with HT-DNA for 4 h. (G) ELISA analysis of IFN-β protein levels in sh-control and sh-METTL3 A549 cells treated with HT-DNA for 4 h. (H) The IFNB1 promoter activities in sh-control and sh-METTL3 A549 cells treated with HT-DNA for 4 h. (I) The mRNA levels of *IFNB1* and *CXCL0* in sh-control and sh-METTL3 A549 cells treated with G140 or H151 for 4 h. (J) ELISA analysis of IFN-β protein levels in sh-control and sh-METTL3 A549 cells treated with G140 or H151 for 4 h. (K to M) The tumor growth curves (K), the tumor volume (L), and the tumor weight (M) of syngeneic tumor models using sh-control and sh-*METTL3* LLC cells with or without G140 treatment. (N and O) The percentages of CD45^+^ cells (N) and the percentages of CD8^+^ T cells in CD45^+^ cells (O) in the tumor tissues taken from mice with sh-control and sh-METTL3 LLC syngeneic tumor with or without G140 treatment. (P) IHC (METTL3, CD8, and Granzyme B [GZMB])-stained paraffin-embedded sections obtained from sh-control and sh-*METTL3* LLC syngeneic tumor with or without G140 treatment; the scale bar is 100 μm. Data are presented as mean ± SD from 3 independent experiments. *P < 0.05, **P < 0.01, and ***P < 0.001, by Student’s *t* test between 2 groups and by one-way ANOVA followed by Bonferroni test for multiple comparison.

To elucidate the mechanism underlying by which METTL3 regulates immune cell infiltration, we established sh-control and sh-METTL3 A549 and H1299 cell lines using lentiviruses (Fig. S2A). Given that cytoplasmic DNA is a key activator of the innate immune response [27], we investigated DNA damage and cytosolic DNA accumulation. To quantify cytosolic DNA, we utilized PicoGreen dye, a fluorescent stain that specifically binds to double-stranded DNA. Notably, under basal conditions, sh-METTL3 A549 cells exhibited substantially higher cytosolic DNA accumulation compared to sh-control cells (Fig. 2A). This accumulation was substantially increased following treatment with Zeocin, a radiomimetic agent that predominantly induces double-strand breaks (DSBs) [28] (Fig. 2B), or exposure to 10 Gy of ionizing radiation (Fig. S2B). To confirm the essential roles of m^6^A in DNA damage and cytosolic DNA accumulation, A549 cells were transfected with wild-type (WT) METTL3 and the catalytically inactive METTL3 mutant (DA, D395A) [29] (Fig. S2C). The results demonstrated that overexpression of WT METTL3, but not the METTL3 DA mutant, reversed the cytosolic DNA accumulation in A549 cells (Fig. 2C). Collectively, these findings suggest that METTL3 deficiency triggers the release of DNA from the nucleus to the cytoplasm in an m^6^A-dependent manner.

To determine whether the increase in cytosolic DNA in sh-*METTL3* cells activates the cGAS pathway, we quantified cGAMP levels in sh-control and sh-*METTL3* LUAD cells. The results showed that sh-METTL3 LUAD cells exhibited significantly higher cGAMP levels compared to sh-control cells, regardless of Zeocin treatment. Moreover, Zeocin treatment further stimulated cGAMP production (Fig. 2D and Fig. S2D). Given that cGAMP activates STING and its downstream factors, the elevated cGAMP levels in sh-*METTL3* A549 cells were accompanied by increased phosphorylation of STING and STAT1 (Fig. 2E). Since the interferon pathway is another critical downstream effector of cGAS activation, we assessed the expression of related genes. We found that the herring testes DNA (HT-DNA)-induced expression of *IFNB1*, as well as that of *CXCL10*, was significantly up-regulated in sh-*METTL3* A549 cells compared to control cells (Fig. 2F). Consistent with these findings, ELISA results confirmed the increased secretion of IFN-β protein (Fig. 2G). Furthermore, the luciferase reporter assay using pGL3-Basic*-IFNB1*-luc demonstrated that *IFNB1* promoter activity was markedly enhanced in sh-*METTL3* A549 cells (Fig. 2H). Similar results were obtained following treatment with STM2457, a potent and selective inhibitor of METTL3/14 [30] (Fig. S2E and F). Collectively, these findings demonstrate that METTL3 knockdown activates the cGAS–STING pathway and interferon responses.

The innate immune signaling triggered by METTL3 deficiency is dependent on cGAS and STING, as the METTL3-deficiency-induced up-regulation of interferon genes and IFN-β protein levels were abolished by treatment with either the cGAS inhibitor G140 [31] or the STING inhibitor H151 [32] (Fig. 2I and J). To validate these findings in vivo, we established syngeneic tumor models using sh-control and sh-METTL3 Lewis lung carcinoma (LLC) cells. The results showed that METTL3 knockdown significantly inhibited the growth of LLC syngeneic tumors, an effect that was rescued by G140 treatment (Fig. 2K). At the experimental endpoint, both tumor volume (Fig. 2L) and weight (Fig. 2M) were pronouncedly lower in the sh-*METTL3* group compared to the sh-control group, and these reductions were reversed by G140 treatment. No significant change in body weight was observed among the groups (Fig. S2G). Furthermore, flow cytometry analysis (Fig. S2H) demonstrated that METTL3 knockdown significantly increased the proportions of CD45^+^ cells and CD8^+^ T cells within the CD45^+^ population, whereas G140 treatment reversed these effects (Fig. 2N and O). Additionally, IHC analysis revealed that sh-*METTL3* LLC syngeneic tumors exhibited high expression levels of CD8 and Granzyme B (GZMB) compared to control tumors (Fig. 2P). In summary, these data indicate that METTL3 deficiency promotes nuclear DNA leakage into the cytoplasm, thereby triggering antitumor immunity through the activation of the cGAS pathway.

### METTL3 regulates cytosolic DNA levels by modulating homologous recombination repair efficiency

**Fig. 3.**
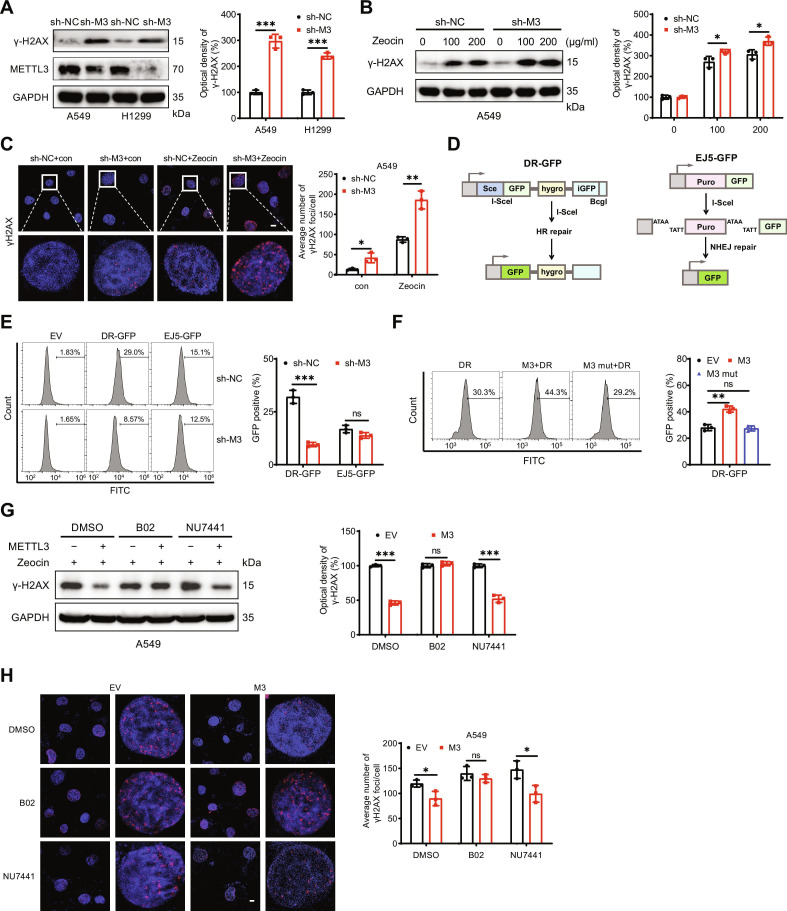
METTL3 regulates cytosolic DNA levels by modulating homologous recombination (HR) efficiency. (A) The protein expression of γH2AX in sh-control and sh-METTL3 A549 and H1299 cells (left) and quantitative analysis (right). (B) The protein expression of γH2AX in sh-control and sh-METTL3 A549 with Zeocin treatment (left) and quantitative analysis (right). (C) Representative confocal images of γH2AX in sh-control and sh-METTL3 A549 cells with or without Zeocin treatment; scale bar = 10 μm. (D) Schematic of the plasmid system for determining the frequency of HR- or NHEJ-mediated double-strand break repair (DSBR). (E) Quantification of the frequency of HR-mediated and NHEJ-mediated DSBR in sh-control and sh-METTL3 A549 cells. EV, empty vector. (F) Quantification of the frequency of HR-mediated DSBR in A549 cells transfected with vector control, METTL3 WT plasmid, and METTL3 DA mutant plasmid for 24 h. (G) The protein expression of γH2AX in A549 cells transfected with vector control or METTL3 WT plasmid and pretreatment with B02 (a specific inhibitor of RAD51 and HR), NU7441 (a specific inhibitor of ligase IV and NHEJ), or dimethyl sulfoxide (DMSO) and Zeocin treatment for 4 h. (H) Representative confocal images of γH2AX in A549 cells transfected with vector control or METTL3 WT plasmid and pretreatment with B02 (a specific inhibitor of RAD51 and HR), NU7441 (a specific inhibitor of ligase IV and NHEJ), or DMSO and Zeocin treatment for 4 h; scale bar = 10 μm. Data are presented as mean ± SD from 3 independent experiments. *P < 0.05, **P < 0.01, ***P < 0.001, and ns, no significance, by Student’s *t* test between 2 groups and by one-way ANOVA followed by Bonferroni test for multiple comparison.

Given that the accumulation of cytoplasmic DNA can result from nuclear DNA damage, we assessed the phosphorylation of histone H2AX (γH2AX), a surrogate marker for DNA DSBs, in LUAD cells. Western blotting analysis revealed that METTL3 knockdown significantly increased γH2AX level in A549 and H1299 cells (Fig. 3A). Furthermore, γH2AX levels remained elevated in sh-*METTL3* A549 cells and were markedly enhanced following Zeocin treatment (Fig. 3B). Consistently, confocal microscopy confirmed elevated γH2AX levels in Zeocin-treated sh-METTL3 A549 cells (Fig. 3C). These results suggest that METTL3 deficiency leads to delayed repair and/or persistent generation of DSBs.

DSBs are primarily repaired by error-free homologous recombination (HR) or error-prone nonhomologous end joining (NHEJ) repair pathways [33]. To determine whether METTL3 influences one or both pathways, we utilized the HR and NHEJ repair reporter systems (Fig. 3D). The results indicated that METTL3 deficiency impaired HR-mediated DSB repair (DSBR) but did not affect NHEJ-mediated DSBR (Fig. 3E). Moreover, overexpression of WT METTL3, but not the METTL3 DA mutant, significantly enhanced HR-mediated DSBR (Fig. 3F). These findings suggest that METTL3 regulates HR efficiency in a methyltransferase-activity-dependent manner. Furthermore, in the presence of B02, a specific inhibitor of RAD51 and HR repair [34], overexpression of METTL3 failed to stimulate the repair of Zeocin-induced DSBs. Conversely, NU7441, a specific inhibitor of DNA ligase IV and NHEJ repair [35], did not impair METTL3-stimulated repair of Zeocin-induced DSBs (Fig. 3G and H). Collectively, these findings indicate that METTL3 knockdown promotes cytosolic DNA accumulation by reducing the efficiency of HR-mediated DSBR.

### MSH5 mediates METTL3-regulated HR repair efficiency and cytosolic DNA accumulation

**Fig. 4.**
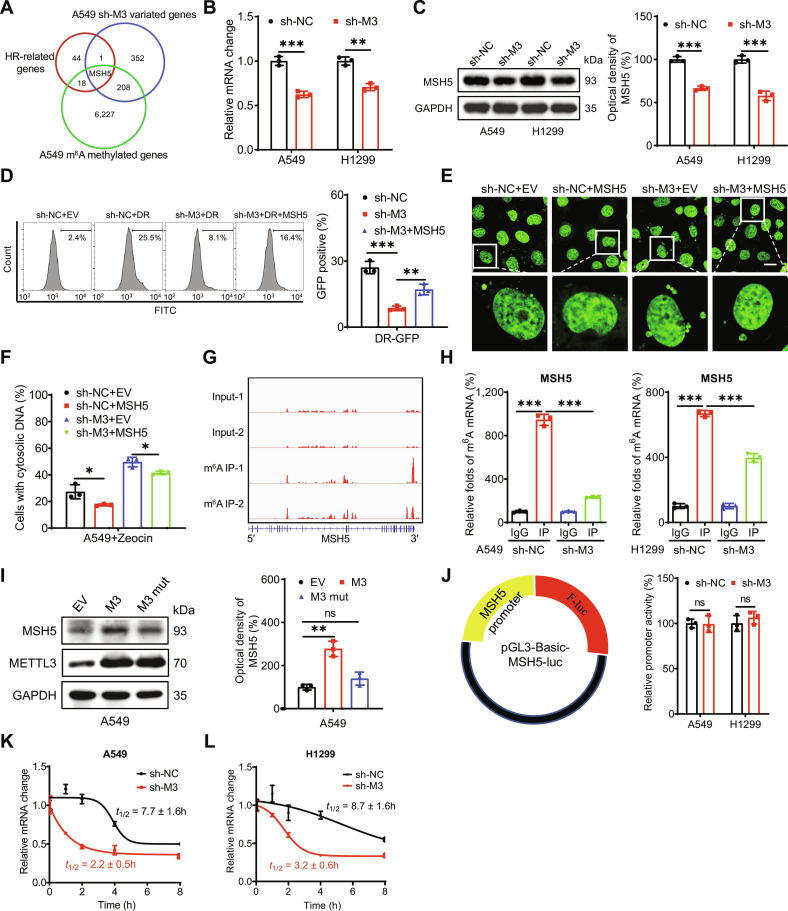
MSH5 mediates METTL3-regulated HR efficiency and cytosolic DNA accumulation. (A) The Venn diagram shows substantial overlap among HR-related genes, variated genes between sh-METTL3 and sh-control A549 cells, and *N^6^*-methyladenosine (m^6^A)-modified genes in A549 cells. (B and C) The mRNA (B) and protein (C) expressions of *MSH5* in sh-control and sh-*METTL3* A549 and H1299 cells. (D) Quantification of the frequency of HR-mediated DSBR in A549 cells transfected with vector control or MSH5 plasmid for 24 h. (E and F) Representative confocal images of PicoGreen stain in sh-control and sh-METTL3 A549 cells transfected with vector control or MSH5 plasmid (E), and the percentages of cells displaying cytosolic DNA were measured (F); scale bar = 20 μm. (G) m^6^A peaks were enriched in *MSH5* mRNA from m^6^A RIP-seq data. (H) m^6^A RIP-qPCR analysis of *MSH5* in sh-control and sh-*METTL3* A549 (G) and H1299 (H) cells. (I) The protein expression of MSH5 in A549 cells transfected with vector control, METTL3 WT plasmid, or METTL3 DA mutant plasmid for 24 h. (J) Cells were transfected with pGL3-Basic-*MSH5*-luc reporter and pRL-TK plasmid for 24 h, and the promoter activities were presented as the ratios of the reporter normalized to pRL-TK plasmid. (K and L) After treatment with Act-D for the indicated times, the mRNA levels of *MSH5* were checked in sh-control and sh-*METTL3* A549 (K) and H1299 (L) cells. Data are presented as mean ± SD from 3 independent experiments. *P < 0.05, **P < 0.01, ***P < 0.001, and ns, no significance, by Student’s *t* test between 2 groups and by one-way ANOVA followed by Bonferroni test for multiple comparison.

To identify potential targets involved in m^6^A-regulated HR repair efficiency and cytosolic DNA accumulation, we performed RNA sequencing on sh-control and sh-*METTL3* A549 cells. We identified 562 differentially expressed genes in sh-METTL3 A549 cells, including 333 up-regulated and 229 down-regulated genes (Fig. S3A; Table S1). Among 64 HR-related genes (Table S2), *MSH5* (mutS homolog 5) was the only gene to exhibit a greater than 2.0-fold change in expression (*P* < 0.05) in sh-METTL3 A549 cells compared to sh-control cells. Notably, *MSH5* was also identified as an m^6^A-modified gene in A549 cells via m^6^A-seq (Table S3 and Fig. 4A). Consistent with this, RT-qPCR confirmed that METTL3 knockdown decreased *MSH5* mRNA levels in both A549 and H1299 cells (Fig. 4B). Furthermore, Western blot analysis demonstrated that METTL3 knockdown reduced MSH5 protein expression in both cell lines (Fig. 4C).

*MSH5* encodes a member of the mutS family, which plays a critical role in DNA mismatch repair and meiotic recombination [36]. To investigate the involvement of MSH5 in METTL3-regulated HR repair efficiency and cytosolic DNA accumulation in LUAD cells, we performed rescue experiments. Our results demonstrated that MSH5 overexpression (Fig. S3B) reversed the reduction in HR repair efficiency (Fig. 4D) and attenuated the increase in cytosolic DNA accumulation (Fig. 4E and F) in sh-*METTL3* A549 cells. Collectively, these findings indicate that MSH5 mediates the effects of METTL3 on HR repair efficiency and cytosolic DNA accumulation in LUAD cells.

We further investigated whether m^6^A regulates MSH5 expression through direct mRNA methylation. m^6^A-RIP-seq data revealed that the coding sequence (CDS) region of *MSH5* mRNA undergoes m^6^A modification (Fig. 4G). Additionally, m^6^A-RIP-qPCR confirmed a 10-fold enrichment of *MSH5* mRNA using the m^6^A antibody in *MSH5* mRNA in A549 cells, which was significantly reduced in sh-*METTL3* A549 and H1299 (Fig. 4H) cells. Furthermore, overexpression of WT METTL3, but not the METTL3 DA mutant, rescued MSH5 level in A549 cells (Fig. 4I). Collectively, these results demonstrate that MSH5 was modified by m^6^A and that m^6^A positively regulates MSH5 expression in LUAD cells.

To investigate the underlying mechanisms of m^6^A-regulated MSH5 expression, we first examined whether METTL3 modulates MSH5 transcription. The luciferase reporter assay by transfecting the promoter reporter plasmid pGL3-Basic-*MSH5*-luc into A549 cells revealed no significant difference in *MSH5* promoter activity between sh-control and sh-*METTL3* LUAD cells (Fig. 4J), suggesting that METTL3 does not affect MSH5 transcription*.* This finding was further corroborated by qRT-PCR analysis, which showed comparable levels of *MSH5* precursor mRNA in sh-control and sh-*METTL3* LUAD cells (Fig. S3C). Additionally, subcellular fractionation assays revealed no difference in the distribution of *MSH5* mRNA between sh-control and sh-*METTL3* A549 cells (Fig. S3D). These findings indicated that m^6^A modification does not influence the subcellular localization of *MSH5* mRNA*.*

However, METTL3 knockdown (Fig. 4B) significantly reduced *MSH5* mRNA levels in LUAD cells. Given that m^6^A did not affect promoter activity but did regulate*MSH5* mRNA abundance, we investigated its effect on mRNA stability. Our results showed that METTL3 knockdown substantially decreased the stability of *MSH5* mRNA in both A549 (Fig. 4K) and H1299 (Fig. 4L) cells. Thus, MSH5 expression is positively regulated by m^6^A through the modulation of mRNA stability.

Regarding the translation efficiency of endogenous *MSH5* mRNA, which is defined as the ratio of protein production (MSH5/GAPDH) to mRNA abundance [37], METTL3 knockdown had no significant impact on MSH5 translation efficiency in A549 cells (Fig. S3E). To determine whether m^6^A influences MSH5 posttranslationally, sh-control and sh-*METTL3* A549 cells were treated with cycloheximide to block protein synthesis. Our data revealed that the half-life of the MSH5 protein was comparable between sh-control and sh-*METTL3* cells (Fig. S3F). Collectively, these data indicated that METTL3 positively regulates *MSH5* mRNA stability without affecting its transcription, nuclear export, translation efficiency, or protein stability.

### METTL3 stabilizes *MSH5* mRNA by recruiting IGF2BP2 to the m^6^A site A2521

**Fig. 5.**
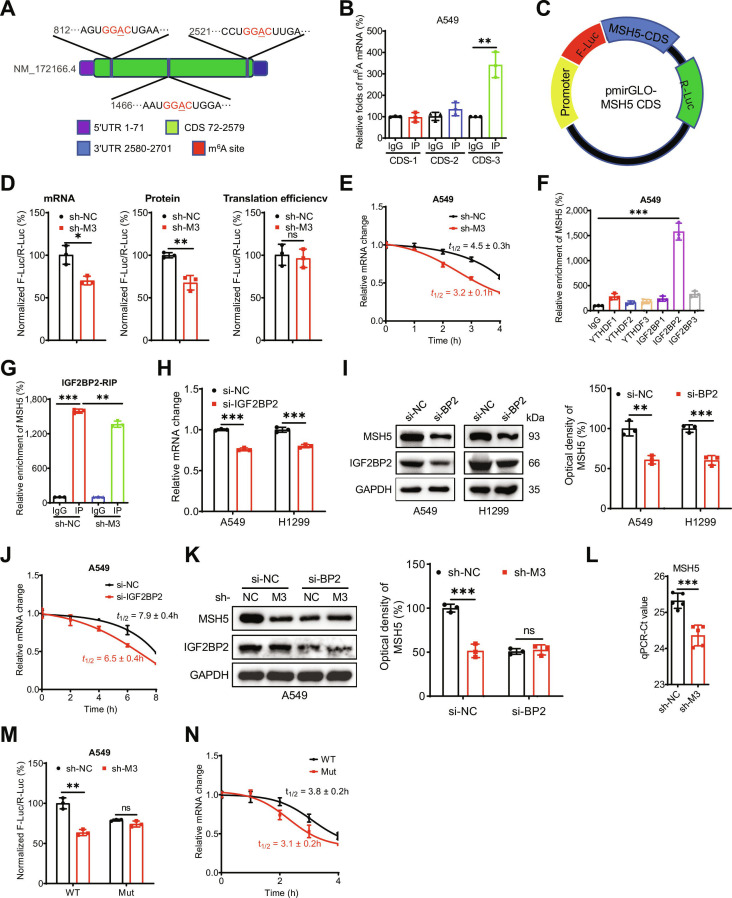
METTL3 stabilizes *MSH5* mRNA by recruiting IGF2BP2 to the m^6^A site A2521. (A) Schematic representation of the 3 GGAC motifs in MSH5 CDS. (B) The m^6^A of the 3 GGAC motifs in MSH5 CDS in A549 cells was analyzed by m^6^A-RIP-qPCR using fragmented RNA. (C) Schematic representation of pmirGLO-*MSH5* CDS reporter. (D) The mRNA abundance, luciferase activity, and translation efficiency of F-Luc in sh-control and sh-*METTL3* A549 cells transfected with pmirGLO-*MSH5* CDS reporter for 24 h. (E) After treatment with Act-D for the indicated times, the mRNA levels of *F-Luc* were checked in sh-control and sh-*METTL3* A549 cells transfected with pmirGLO-*MSH5* CDS reporter for 24 h. (F) RIP-qPCR analysis of *MSH5* mRNA in A549 cells by use of antibody of YTHDF1-3 and IGF2BP1-3. (G) IGF2BP2 RIP-qPCR analysis of *MSH5* mRNA in sh-control and sh-*METTL3* A549 cells. (H and I) The mRNA levels (H) and protein expression (I) of MSH5 in A549 and H1299 cells transfected with si-NC or si-IGF2BP2 for 24 h. (J) After treatment with Act-D for the indicated times, the mRNA levels of *MSH5* were checked in A549 cells transfected with si-NC or si-IGF2BP2 for 24 h. (K) The protein expression of MSH5 in sh-control and sh-*METTL3* A549 cells transfected with si-NC or si-IGF2BP2 for 24 h. (L) The threshold cycle (Ct) of qPCR showing SELECT results for detecting the m^6^A site in the potential m^6^A site of MSH5 in sh-control and sh-*METTL3* A549 cells. (M) The relative luciferase activity of F-Luc/R-Luc of pmirGLO-*MSH5* CDS WT and Mut reporter in sh-control and sh-*METTL3* A549 cells. (N) After treatment with Act-D for the indicated times, the mRNA levels of *F-Luc* were checked in sh-control and sh-*METTL3* A549 cells transfected with pmirGLO-*MSH5* CDS WT and Mut reporter for 24 h. Data are presented as mean ± SD from 3 independent experiments. *P < 0.05, **P < 0.01, ***P < 0.001, and ns, no significance, by Student’s *t* test between 2 groups and by one-way ANOVA followed by Bonferroni test for multiple comparison.

We further investigated the methylation site and the reader protein responsible for m^6^A-mediated stabilization of *MSH5* mRNA. Based on the m^6^A-seq results (Fig. 4F) and *MSH5* mRNA m^6^A site predictions using SRAMP (http://www.cuilab.cn/sramp) (Fig. S4A), we identified 3 GGAC motifs in the *MSH5* CDS (Fig. 5A). m^6^A-RIP-PCR performed on fragmented poly(A)^+^ RNA indicated that the relative enrichment of the *MSH5* CDS-3 was significantly higher than that of the other 2 CDSs in A549 cells (Fig. 5B). To determine whether m^6^A modification of CDS-3 contributes to the regulation of *MSH5* mRNA stability, we constructed CDS reporters by inserting the WT *MSH5* CDS downstream of the firefly luciferase reporter gene in the pmirGLO vector (Fig. 5C). Luciferase assay revealed that F-Luc levels were markedly decreased in sh-*METTL3* A549 cells due to the down-regulation of *F-Luc* mRNA, rather than a decrease in translation efficiency (Fig. 5D). Furthermore, the presence of the *MSH5* CDS reduced the half-life of *F-Luc* mRNA in sh-*METTL3* A549 cells (Fig. 5E), confirming that the m^6^A-methylated CDS mediated the METTL3-regulated *MSH5* mRNA stability.

The m^6^A-binding proteins, insulin-like growth factor 2 mRNA-binding proteins (IGF2BPs; including IGF2BP1/2/3), but not the YTH domain family proteins YTHDF1/2/3, recognize and stabilize m^6^A-modified cellular RNAs [38,39]. RIP-qPCR revealed that IGF2BP2, but not IGF2BP1/3 or YTHDF1/2/3, binds to *MSH5* mRNA in A549 cells (Fig. 5F). Furthermore, the interaction between IGF2BP2 and *MSH5* mRNA was diminished in sh-*METTL3* A549 cells (Fig. 5G). Knockdown of IGF2BP2 expression suppressed both the mRNA (Fig. 5H) and protein (Fig. 5I) levels of MSH5 in A549 and H1299 cells. This effect was attributed to decreased *MSH5* mRNA stability following IGF2BP2 knockdown (Fig. 5J). Additionally, we found that si-IGF2BP2 reduced MSH5 expression and attenuated the reduction in MSH5 expression mediated by METTL3 deletion in A549 cells (Fig. 5K). Collectively, these data suggest that IGF2BP2 mediates the m^6^A-dependent regulation of MSH5 expression.

Our data suggested that A2521 within CDS-3 might be a potential m^6^A site on *MSH5* mRNA*.* We further validated this site by the “SELECT” method [40] in A549 cells. The results revealed that METTL3 knockdown significantly reduced the methylation level of A2521 (Fig. 5L). In contrast, the nearby non-m^6^A-modified nucleotide A2526 exhibited a notably lower threshold cycle value compared to A2521 (Fig. S4B). To investigate the role of A2521 in m^6^A-regulated *MSH5* mRNA stability, we mutated this site within the pmirGLO-*MSH5* CDS reporter (Fig. S4C). The A2521 mutation resulted in decreased F-Luc activity (Fig. 5M), whereas the reduction was reversed in sh-*METTL3* cells. Furthermore, mutation of A2521 decreased the stability of *F-Luc* mRNA (Fig. 5N). Collectively, these data confirm that A2521 within the *MSH5* CDS mediates the m^6^A-regulated stability of *MSH5* mRNA.

### METTL3 destabilizes *cGAS* mRNA by recruiting YTHDF2 and methylating A1545

**Fig. 6.**
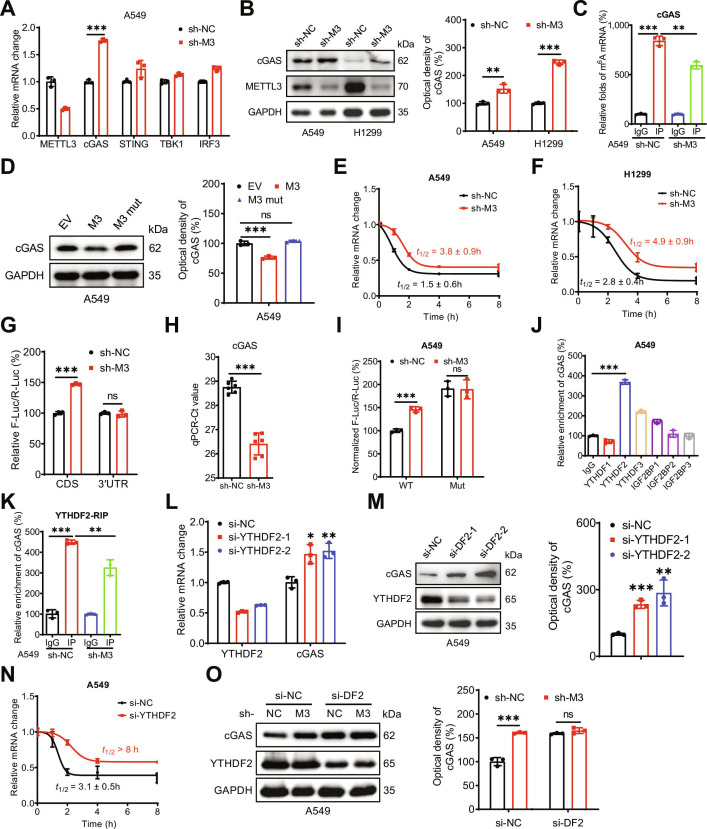
METTL3 destabilizes *cGAS* mRNA by recruiting YTHDF2 and methylating A1545. (A) The mRNA expression of cGAS, STING, TBK1, and IRF3 in sh-control and sh-METTL3 A549 cells. (B) The protein expression of cGAS in sh-control and sh-METTL3 A549 and H1299 cells. (C) m^6^A RIP-qPCR analysis of cGAS in sh-control and sh-*METTL3* A549 cells. (D) The protein expression of cGAS in A549 cells transfected with vector control, METTL3 WT plasmid, or METTL3 DA mutant plasmid for 24 h. (E) After treatment with Act-D for the indicated times, the mRNA levels of cGAS were checked in sh-control and sh-*METTL3* A549 cells. (F) After treatment with Act-D for the indicated times, the mRNA levels of cGAS were checked in sh-control and sh-*METTL3* H1299 cells. (G) The relative luciferase activity of F-Luc/R-Luc of pmirGLO-cGAS-CDS reporter or pmirGLO-cGAS-5′UTR reporter in sh-control and sh-*METTL3* A549 cells. (H) The Ct of qPCR showing SELECT results for detecting the m^6^A site in the potential m^6^A site of cGAS in sh-control and sh-*METTL3* A549 cells. (I) The relative luciferase activity of F-Luc/R-Luc of pmirGLO-cGAS-CDS WT and Mut reporter in sh-control and sh-*METTL3* A549 cells. (J) RIP-qPCR analysis of cGAS mRNA in A549 cells by use of antibody of YTHDF1-3 and IGF2BP1-3. (K) YTHDF2 RIP-qPCR analysis of cGAS mRNA in sh-control and sh-*METTL3* A549 cells. (L) The mRNA levels of cGAS in A549 cells transfected with si-NC or si-YTHDF2-1/2 for 24 h. (M) The protein expression of cGAS in A549 cells transfected with si-NC or si-YTHDF2-1/2 for 24 h. (N) After treatment with Act-D for the indicated times, the mRNA levels of cGAS were checked in sh-control and sh-*METTL3* A549 cells transfected with si-NC or si-YTHDF2 for 24 h. (O) The protein expression of cGAS in sh-control and sh-*METTL3* A549 cells transfected with si-NC or si-YTHDF2 for 24 h. Data are presented as mean ± SD from 3 independent experiments. *P < 0.05, **P < 0.01, ***P < 0.001, and ns, no significance, by Student’s *t* test between 2 groups and by one-way ANOVA followed by Bonferroni test for multiple comparison.

To further elucidate the role of METTL3 in modulating the innate immune response, we examined its effects on key components of the cGAS–STING pathway. RT-qPCR analysis revealed that METTL3 knockdown significantly up-regulated *cGAS* mRNA expression in both A549 (Fig. 6A) and H1299 (Fig. S5A) cells. Consistent with these findings, Western blot analysis demonstrated a corresponding increase in cGAS protein levels upon METTL3 knockdown in LUAD cells (Fig. 6B). These results suggest that METTL3 knockdown may positively regulate the innate immune response by modulating cGAS expression.

We further investigated whether m^6^A regulates cGAS expression through direct mRNA methylation. m^6^A-RIP-qPCR confirmed that the enrichment of *cGAS* mRNA by the m^6^A antibody was 8-fold higher in A549 cells, whereas this enrichment was significantly reduced in sh-*METTL3* A549 (Fig. 6C) and H1299 (Fig. S5B) cells. These results indicated that *cGAS* mRNA undergoes m^6^A modification. Moreover, transfection of WT METTL3, but not the METTL3 DA mutant, decreased cGAS expression in A549 cells (Fig. 6D). Additionally, treatment with STM2457 pronouncedly increased cGAS protein levels in A549 cells (Fig. S5C). Collectively, these findings demonstrate that METTL3 negatively regulates cGAS expression in LUAD cells in an m^6^A-dependent manner.

We further investigated the mechanisms underlying the m^6^A-regulated expression of cGAS. Our data indicated that the transcription (Fig. S5D), subcellular localization (Fig. S5E), and translation efficiency (Fig. S5F) of *cGAS* mRNA, as well as the half-life of the cGAS protein (Fig. S5G), were comparable between sh-control and sh-METTL3 cells. We then examined mRNA stability. The results revealed that METTL3 knockdown significantly increased the mRNA stability of *cGAS* in both A549 (Fig. 6E) and H1299 (Fig. 6F) cells. Collectively, these data suggest that METTL3 negatively regulates *cGAS* mRNA stability without affecting its transcription, nuclear export, translation efficiency, or protein stability.

Furthermore, we investigated the specific methylation site and reader protein responsible for the m^6^A-mediated regulation of *cGAS* mRNA stability. As shown in Fig. S5H, 3 GGAC motifs were identified in the *cGAS* mRNA sequence by the m^6^A site predictor. To determine whether m^6^A modifications within the CDS or 3′ untranslated region (UTR) contribute to the regulation of cGAS, we constructed CDS and 3′UTR reporters by inserting the WT cGAS CDS or 3′UTR downstream of the firefly luciferase gene in the pmirGLO vector (Fig. S5I). Luciferase assays revealed a substantial increase in F-Luc activity from the pmirGLO-cGAS-CDS reporter, but not from the pmirGLO-cGAS-3′UTR reporter, in sh-*METTL3* A549 cells (Fig. 6G). Additionally, m^6^A of A1545 within the GGAC motif in the *cGAS* CDS was validated by the “SELECT” method in A549 cells, whereas METTL3 knockdown markedly reduced the methylation level at A1545 (Fig. 6H). The nearby non-m^6^A-modified nucleotide A1542 exhibited a pronouncedly lower threshold cycle value compared to A1545 (Fig. S5J). Moreover, mutation of A1545 in the CDS increased F-Luc activity, which was abolished in sh-*METTL3* cells (Fig. 6I). Collectively, these data confirm that methylation of A1545 within the *cGAS* CDS is critical for the m^6^A-dependent regulation of cGAS.

We further investigated the reader protein responsible for the m^6^A-regulated expression of cGAS. RIP-qPCR revealed that YTHDF2, but not IGF2BP1/2/3 or YTHDF1/3, binds to *cGAS* mRNA (Fig. 6J). Furthermore, the interaction between YTHDF2 and *cGAS* mRNA was diminished in sh-*METTL3* A549 cells (Fig. 6K). Knockdown of YTHDF2 increased both the mRNA (Fig. 6L) and protein (Fig. 6M) expression of cGAS in A549 cells. This effect was attributed to the increased stability of *cGAS* mRNA following YTHDF2 depletion (Fig. 6N). Additionally, we found that si-YTHDF2 increased cGAS expression and attenuated the up-regulation in sh-METTL3-induced cGAS expression in A549 cells (Fig. 6O). Collectively, these data suggest that YTHDF2 mediates the m^6^A-regulated expression of cGAS.

### METTL3 inhibition enhances LUAD immunotherapy efficacy and suppresses cancer progression

**Fig. 7.**
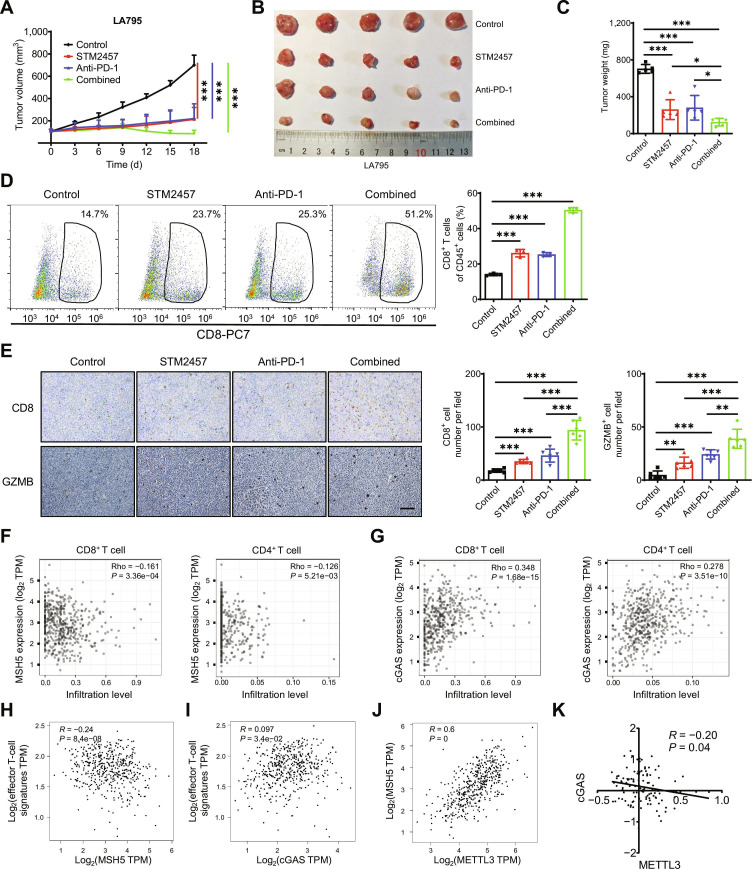
METTL3 inhibition enhances immunotherapy and suppresses cancer progression in LUAD. (A to C) The tumor growth curves (A), the tumor volume (B), and the tumor weight (C) of syngeneic tumor models using LA795 cells with STM2457, anti-PD-1 antibody, or a combination of both treatments. (D) The percentages of CD8^+^ T cells in CD45^+^ cells in the tumor tissues taken from mice with LA795 syngeneic tumors with STM2457, anti-PD-1 antibody, or a combination of both treatments. (E) IHC (CD8 and GZMB)-stained paraffin-embedded sections obtained from LA795 syngeneic tumor with STM2457, anti-PD-1 antibody, or a combination of both treatments; the scale bar is 100 μm. (F) The correlation between the expression of MSH5 and the infiltrating level of CD8^+^ T cells or CD4^+^ T cells in LUAD based on the TIMER platform. (G) The correlation between the expression of cGAS and the infiltrating level of CD8^+^ T cells or CD4^+^ T cells in LUAD based on the TIMER platform. (H) The correlation between the expression of MSH5 and effector T-cell markers (including CX3CR1, FGFBP2, and FCGR3A) in LUAD based on the TCGA database. (I) The correlation between the expression of cGAS and effector T-cell markers (including CX3CR1, FGFBP2, and FCGR3A) in LUAD based on the TCGA database. (J) The correlation between the expression of METTL3 and MSH5 in LUAD based on the TCGA database. (K) The correlation between the expression of METTL3 and cGAS in LUAD from the CPTAC database. Data are presented as mean ± SD from 3 independent experiments. *P < 0.05, **P < 0.01, and ***P < 0.001 by Student’s *t* test between 2 groups and by one-way ANOVA followed by Bonferroni test for multiple comparison.

To evaluate the therapeutic potential of combining METTL3 inhibition with immunotherapy, we established syngeneic tumor models using LA795 cells treated with STM2457, anti-PD-1 antibody, or their combination. The results demonstrated that METTL3 inhibition significantly improved the efficacy of anti-PD-1 antibody therapy, resulting in marked reductions in tumor volume and weight (Fig. 7A to C). Subsequently, flow cytometry analysis demonstrated that the combination of METTL3 inhibition and anti-PD-1 antibody treatment notably increased the proportion of CD8^+^ T cells within the tumor tissue (Fig. 7D). IHC analysis confirmed elevated expression of CD8 and GZMB following the combination treatment (Fig. 7E). Collectively, these findings indicate that METTL3 inhibition enhances tumor immune infiltration and improves the efficacy of immunotherapy. Similar results were observed in LLC syngeneic tumor models (Fig. S6A to E).

To further investigate the potential link between METTL3-inhibition-induced activation of the cGAS/STING axis and LUAD progression, we analyzed clinical datasets from public databases. TIMER2.0 data analysis demonstrated that MSH5 expression was significantly negatively correlated with the infiltration of multiple antitumor immune cell types, including CD8^+^ T cells (*r* = −0.161, *P* = 3.36e−04), CD4^+^ T cells (*r* = −0.126, *P* = 5.21e−03), monocytes (*r* = −0.264, *P* = 2.51e−09), and neutrophils (*r* = −0.113, *P* = 1.20e−03) (Fig. 7F and Fig. S6A). In contrast, cGAS expression was positively correlated with the infiltration of these immune cell subsets (CD8^+^ T cells, *r* = 0.348, *P* = 1.68e−15; CD4^+^ T cells, *r* = 0.278, *P* = 3.51e−10; monocytes, *r* = 0.335, *P* = 2.01e−14; and neutrophils, *r* = 0.469, *P* = 2.75e−28, respectively) (Fig. 7G and Fig. S6B). Moreover, the expression levels of effector T-cell markers were inversely correlated with MSH5 expression (*r* = −0.24, *P* = 8.4e−08) (Fig. 7H) but positively correlated with cGAS expression (*r* = 0.097, *P* = 3.4e−02) (Fig. 7I) in LUAD tissues. Compared with normal tissues, MSH5 expression was up-regulated in LUAD tumor tissues within the The Cancer Genome Atlas (TCGA) cohort (Fig. S6C). Furthermore, Gene Expression Profiling Interactive Analysis data revealed a positive correlation between METTL3 and MSH5 expression (Fig. 7J) in LUAD patients. Similarly, METTL3 protein expression was negatively correlated with cGAS (Fig. 7K), CXCL10, and ISG15 (Fig. S6D) in LUAD patient samples from the CPTAC database. Collectively, these data suggest that METTL3-inhibition-induced activation of the cGAS/STING axis modulates the immune microenvironment and suppresses LUAD progression.

### METTL3 inhibition combined with PARP inhibitor enhances the antitumor effects of an anti-PD-1 antibody

**Fig. 8.**
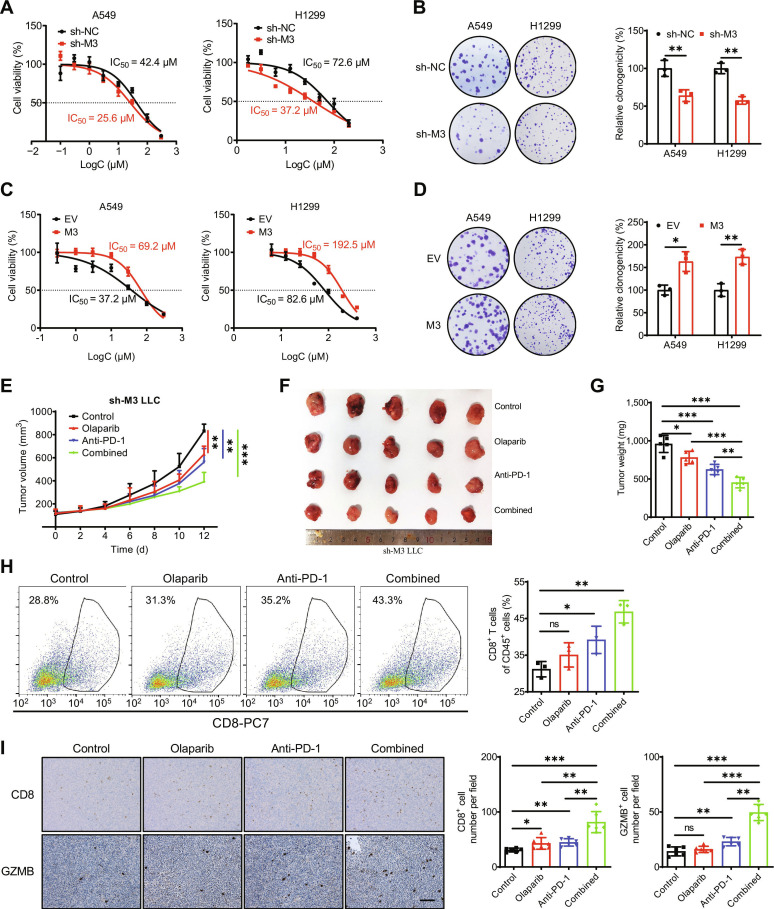
METTL3 inhibition and the PARP inhibitor combined enhance the antitumor functions of the PD-1 antibody. (A) The sensitivity of sh-control or sh-METTL3 A549 and H1299 cells to olaparib. (B) The colony-formation capacity of sh-control or sh-METTL3 A549 and H1299 cells treated with olaparib. (C) The sensitivity of A549 and H1299 cells transfected with vector control and METTL3 WT plasmid for 24 h to olaparib. (D) The colony-formation capacity of A549 and H1299 cells transfected with vector control and METTL3 WT plasmid for 24 h, treated with olaparib. (E) The tumor growth curves of syngeneic tumor models using sh-*METTL3* LLC cells with olaparib, anti-PD-1 antibody, or a combination of both treatments. (F) The tumor volume of using sh-*METTL3* LLC cells with olaparib, anti-PD-1 antibody, or a combination of both treatments at the end of the experiment. (G) The tumor weight of using sh-*METTL3* LLC cells with olaparib, anti-PD-1 antibody, or a combination of both treatments. (H) The percentages of CD8^+^ T cells in CD45^+^ cells in the tumor tissues taken from mice with sh-*METTL3* LLC syngeneic tumor with olaparib, anti-PD-1 antibody, or a combination of both treatments. (I) IHC (CD8 and GZMB)-stained paraffin-embedded sections obtained from sh-*METTL3* LLC syngeneic tumor with olaparib, anti-PD-1 antibody, or a combination of both treatments; the scale bar is 100 μm. Data are presented as mean ± SD from 3 independent experiments. *P < 0.05, **P < 0.01, ***P < 0.001, and ns, no significance, by Student’s *t* test between 2 groups and by one-way ANOVA followed by Bonferroni test for multiple comparison.

Poly(ADP-ribose) polymerase 1 is responsible for initiating the repair of single-strand breaks, and unrepaired single-strand breaks can lead to the formation of detrimental DSBs [41]. Recent studies have shown that PARP inhibitors can induce micronuclei formation in cancer cell lines and trigger the expression of ISGs in a cGAS- and STING-dependent manner [42,43]. Given that we previously observed that METTL3 deficiency impairs HR repair efficacy, thereby activating cGAS/STING-mediated antitumor immunity, we investigated whether METTL3 regulates the response to PARP inhibitors in LUAD cells. Cell viability assays revealed that METTL3 knockdown significantly sensitized A549 and H1299 cells to olaparib, a PARP inhibitor (Fig. 8A). Additionally, colony-formation assay demonstrated that METTL3 knockdown markedly inhibited the clonogenicity of both A549 and H1299 cells (Fig. 8B). Conversely, METTL3 overexpression reduced sensitivity and enhanced colony formation in A549 cells treated with olaparib (Fig. 8C and D). Collectively, these data suggest that METTL3 loss sensitizes tumor cells to PARP inhibitors and impairs oncogenic transformation.

Subsequently, we treated sh-METTL3 LLC syngeneic tumor models with olaparib, an anti-PD-1 antibody, or a combination of both and monitored tumor growth (Fig. 8E). Notably, monotherapy with either the PARP inhibitor or the anti-PD-1 antibody restricted tumor growth and reduced tumor weight, whereas their combination substantially improved the therapeutic effects (Fig. 8F and G). Additionally, flow cytometry analysis revealed that each treatment increased the intratumoral infiltration of CD8^+^ T cells and that this effect was further enhanced by combined treatment (Fig. 8H). IHC analysis demonstrated that CD8 and GZMB expression levels were elevated following the combination treatment (Fig. 8I). Collectively, our results demonstrate that combining METTL3 targeting with PARP inhibitor enhances the antitumor effects of an anti-PD-1 antibody.

## Discussion

**Fig. 9.**
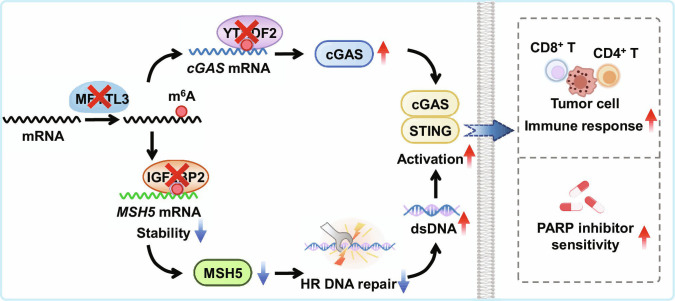
Working model of METTL3 inhibition-activated cGAS/STING axis enhances immunotherapy and PARP inhibitor sensitivity in LUAD. In this model, METTL3 deficiency exacerbates the leakage of nuclear DNA into the cytoplasm, thereby activating the cGAS pathway and enhancing antitumor immunity. Mechanistically, METTL3 deficiency reduces the efficiency of homologous recombination repair by down-regulating MSH5, resulting in elevated levels of cytosolic DNA. The m^6^A methylation at A2521 of *MSH5* mRNA stabilizes its mRNA via binding to IGF2BP2. On the other hand, m^6^A methylation at A1545 within *cGAS* mRNA decreases mRNA stability and modulates its protein expression. Functionally, METTL3 knockdown sensitizes LUAD cells to the PARP inhibitors. Collectively, METTL3 inhibition activates the cGAS/STING-mediated antitumor immunity by inducing cytosolic DNA and cGAS expression, which subsequently regulates the response to PARP inhibitors and cancer progression in LUAD. dsDNA, double-stranded DNA.

While immune checkpoint blockade therapies targeting the PD-1/PD-L1 axis have revolutionized cancer treatment across multiple malignancies [44], their clinical efficacy in LUAD remains limited by immunosuppressive mechanisms and acquired resistance [45]. Thus, there is an urgent need to explore new strategies to improve the efficacy of LUAD immunotherapy. Our study reveals that METTL3-inhibition-induced activation of the cGAS/STING axis enhances immunotherapy efficacy and suppresses LUAD progression by regulating HR repair efficiency and cGAS expression. Mechanistically, IGF2BP2 binds to A2521 in the *MSH5* CDS to increase its mRNA stability, whereas YTHDF2 binds to A1545 in the *cGAS* CDS to decrease its mRNA stability. Overall, METTL3 inhibition activates the cGAS/STING axis, thereby enhancing immunotherapy and PARP-inhibitor sensitivity via the induction of cytosolic DNA and cGAS expression, which in turn regulates LUAD progression (Fig. 9). While previous studies have demonstrated that METTL3 inhibition potentiates immunotherapy responses by enhancing IFN signaling and up-regulating ISGs [46–48], our study elucidates a novel and distinct mechanism by which METTL3 regulates tumor immunogenicity through the cGAS–STING pathway. Importantly, we provide compelling evidence linking METTL3-mediated m^6^A modification to both innate and adaptive immune responses in LUAD, offering new therapeutic opportunities for combination immunotherapy strategies. Collectively, these findings not only substantially advance our mechanistic understanding of METTL3’s role in cancer immunology but also establish a strong preclinical foundation for developing METTL3/cGAS-targeted therapies for LUAD patients.

Extensive research has established the pivotal role of m^6^A modification in regulating cancer development, chemoresistance, and immunotherapy response. The profound influence of m^6^A on immunotherapy efficacy is primarily mediated through its ability to orchestrate the physiological behaviors of immune cells, such as infiltration, survival, differentiation, or polarization, within the tumor microenvironment [7]. For instance, the absence of YTHDF1 enhances the translation efficiency of lysosomal histone proteases in dendritic cells, thereby potentiating the antitumor activity of CD8^+^ T cells [49]. While these findings underscore the importance of m^6^A in adaptive immunity, its role in regulating tumor-intrinsic innate immune signaling remains incompletely understood. Emerging evidence suggests that METTL3 inhibition stimulates an endogenous interferon response in cancer cells through the generation of double-stranded RNA, thereby enhancing antitumor effects [50]. In the present study, we reveal that the knockdown of METTL3 in cancer cells induces cytosolic DNA accumulation and up-regulates cGAS expression, thereby activating the cGAS–STING pathway and amplifying antitumor immunity. Together with prior work, our findings solidify the regulatory role of m^6^A in the innate immune response and provide new mechanistic insights into m^6^A-mediated immune modulation.

METTL3 regulates cytosolic DNA levels by modulating the efficiency of HR repair. Mechanistic investigations revealed that MSH5 mediates METTL3-regulated HR repair efficiency and cytosolic DNA accumulation. MSH5 encodes a member of the mutS family involved in DNA mismatch repair and meiotic recombination [36] and is also considered a crucial component in tumor progression [51,52]. Our data suggest that targeting m^6^A/MSH5-regulated HR repair efficiency could be a promising LUAD therapeutic approach. Additionally, m^6^A modification at A1545 in cGAS decreases its mRNA stability and contributes to m^6^A-regulated cGAS pathway activation in LUAD cells. As the key sensor of accumulated cytosolic DNA, cGAS has emerged as an intriguing candidate for targeted cancer therapy [53,54]. While the posttranscriptional regulation of the cGAS/STING pathway has been extensively studied [20], our study reveals another layer of regulatory control over cGAS expression. Notably, the m^6^A sites on *MSH5* and *cGAS* mRNA are located within their CDS regions but exert opposing effects. A2521 in the *MSH5* CDS binds to IGF2BP2 to increase its mRNA stability, whereas A1545 in the cGAS CDS binds to YTHDF2 to reduce its mRNA stability. This divergence can be attributed to the distinct reader proteins involved, which serve as the functional executors of m^6^A modifications [55].

Our study proposes a combination therapy involving METTL3 inhibitors, PARP inhibitors, and immunotherapy. Nevertheless, it is important to note that targeting METTL3 presents several biological and pharmacological challenges. Given the essential role of m^6^A modification in normal physiological processes, including hematopoiesis and immune cell homeostasis, systemic inhibition could lead to hematologic toxicity or immune dysregulation [56]. While STM2457 demonstrates high biochemical selectivity, potential off-target effects on other methyltransferases (e.g., METTL1 and METTL16) and demethylases (e.g., FTO) remain a concern [57]. Regarding pharmacokinetics, STM2457 is limited by poor tumor penetration and variable bioavailability, particularly in tumors with dense stromal barriers. New-generation inhibitors such as EP652 exhibit improved oral bioavailability and metabolic stability [58]. Furthermore, peptide-based inhibitors that disrupt the METTL3–METTL14 interaction offer a promising strategy with potentially higher specificity and reduced off-target profiles [59]. Thus, while preclinical results are promising, METTL3-targeted therapies have yet to enter clinical evaluation, underscoring the urgent need for early-phase trials to establish the safety, efficacy, and potential of these combination regimens.

In conclusion, our study highlights a novel relationship between the innate immune response and m^6^A modification. Specifically, we demonstrate that METTL3 inhibition activates cGAS/STING-mediated antitumor immunity in LUAD cells by modulating HR repair efficiency and up-regulating cGAS expression. Given the numerous genes involved in the innate immune response, it is plausible that m^6^A modification indirectly regulates the innate immune response by affecting other genes. Additionally, our study indicates that combining METTL3 inhibition with PARP inhibition enhances immunotherapy efficacy, thereby expanding the therapeutic landscape for LUAD patients.

## Materials and Methods

### Human tissue microarray and IHC analysis

This study was approved by the Institutional Ethics Committee of Sun Yat-sen University (No. YBL2024157). Paired tumor and adjacent nontumor paraffin tissue microarray for human LUAD was obtained from YEPCOME Biotechnology Co., Ltd (Shanghai, China). The microarray comprised 50 pairs of LUAD and corresponding nontumor paraffin tissue samples. IHC analysis was performed as previously described [60]. The H-Score was calculated as follows: (percentage of weak intensity cells × 1) + (percentage of moderate intensity cells × 2) + (percentage of strong intensity cells × 3).

### Flow cytometric analysis

Cell-surface-marker staining and flow cytometric analysis for CD3, CD4, CD8, and CD45 expression were performed as described previously [61]. The following antibodies were used: Cy5.5-anti-Mouse CD3e (65-0031-U025; TONBO Bioscience, USA), APC-anti-Mouse CD4 (25-0042-U025; TONBO Bioscience), Cy7-anti-Mouse CD8a (60-0081-U025; TONBO Bioscience), and FITC-anti-Mouse CD45 (35-0451-U025; TONBO Bioscience).

### RNA extraction and real-time PCR

Total RNA was isolated using TRIzol Reagent (Agbio, AG21102, China) and reverse transcribed by Evo M-MLV (Agbio, AG11706). qRT-PCR was assessed with SYBR Green II (Agbio, AG11701) on a CFX Manager 3.1 (Bio-Rad, USA) as recommended by the manufacturer’s protocol. The primers used are listed in Table S4. *GAPDH* was used as a control for normalization. Relative gene expression levels were calculated using the 2^−ΔΔCT^ method.

### Statistical analyses

Data were presented as mean ± SD from a minimum of 3 independent experiments. Statistical analysis was performed using a 2-tailed unpaired Student’s *t* test for comparing 2 groups, while 1-way or 2-way analysis of variance followed by the Bonferroni test was used for multiple comparisons. All statistical tests were 2-sided. Analysis was performed using SPSS 16.0 for Windows. A *P* value of <0.05 was considered to be statistically significant. *P < 0.05, **P < 0.01, and ***P< 0.001; ns, no significant.

Full materials and methods are presented in Supplementary Materials and Methods.

## Data Availability

The data supporting the findings of this study are available within the article and its supplementary materials.
