# Supplementary material for: Methyltransferase-Like 3 Inhibition-Activated cGAS/STING Axis Enhances Immunotherapy and Poly(ADP-Ribose) Polymerase Inhibitor Sensitivity in Lung Adenocarcinoma
Source: Research (Wash D C). 2026 May 20;9:1265. doi: 10.34133/research.1265 (PMC13187501; doi:10.34133/research.1265)
Supplement: Supplementary 1 — Figs. S1 to S6 Tables S1 to S4 [file research.1265.f1.docx]

**Supplementary data for**

**METTL3 inhibition-activated cGAS/STING axis enhances immunotherapy and PARP inhibitor sensitivity in lung adenocarcinoma**

**Zhou et al**


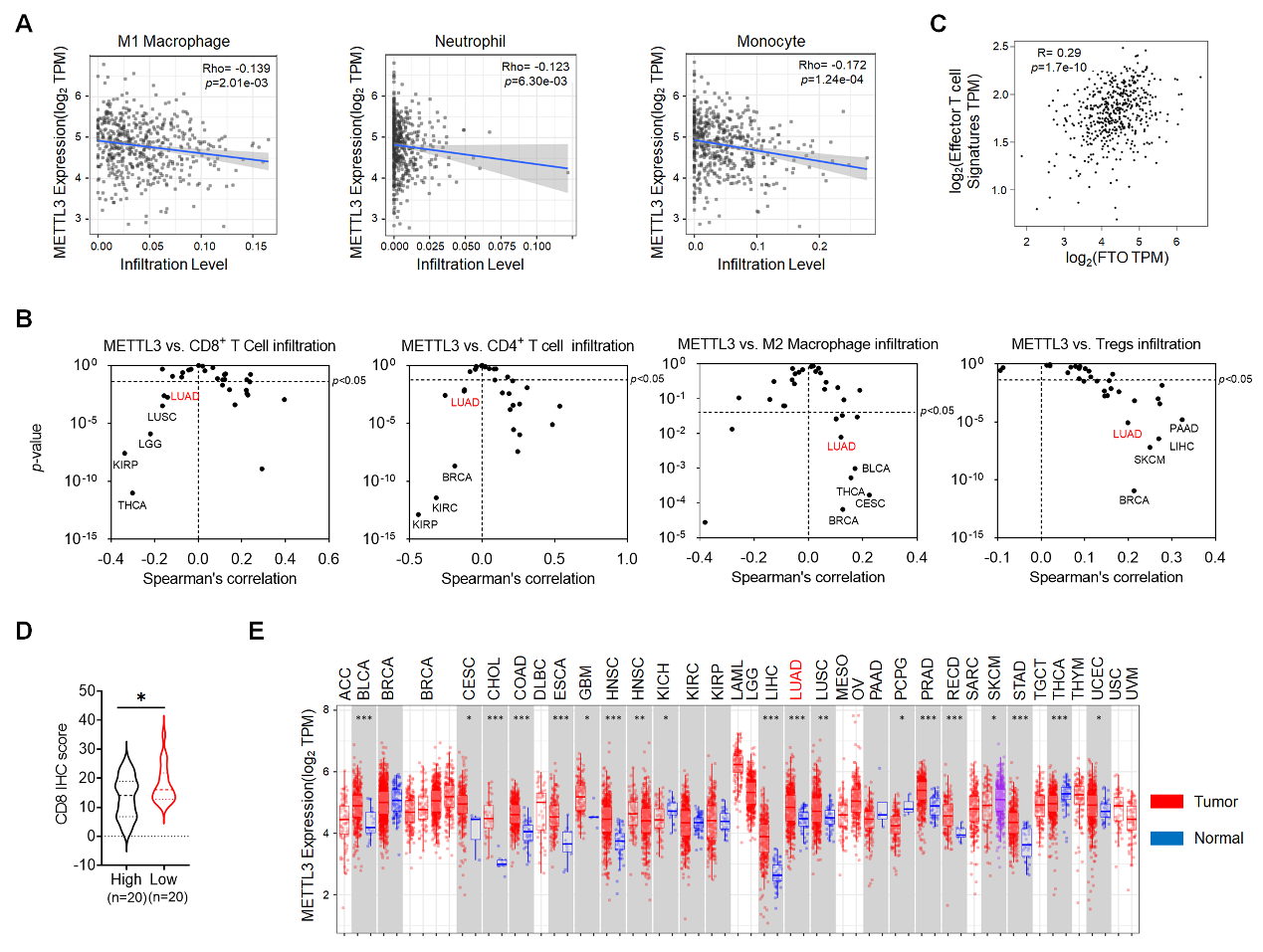


**Supplementary Figure 1 METTL3 expression is correlated with immune cell infiltration and cancer progression.**

1. The correlation between the expression of METTL3 and infiltrating level of M1 macrophages, monocytes, or neutrophils in LUAD based on the TIMER platform;
2. The correlation between the expression of METTL3 and infiltrating level of CD8^+^ T cells, CD4^+^ T cells, M2 macrophages, or Tregs across different types of cancers based on the TIMER platform;
3. The correlation between the expression of FTO and effector T cell markers (including CX3CR1, FGFBP2, FCGR3A) in LUAD based on TCGA database;
4. The IHC scores of CD8 in LUAD tissues with high or low IHC scores of METTL3；
5. The expression of METTL3 across different types of cancers based on the TCGA database.

Data are presented as mean ± SD from three independent experiments. **p*<0.05, by Student’s *t* test between two groups and by one-way ANOVA followed by Bonferroni test for multiple comparisons.

**
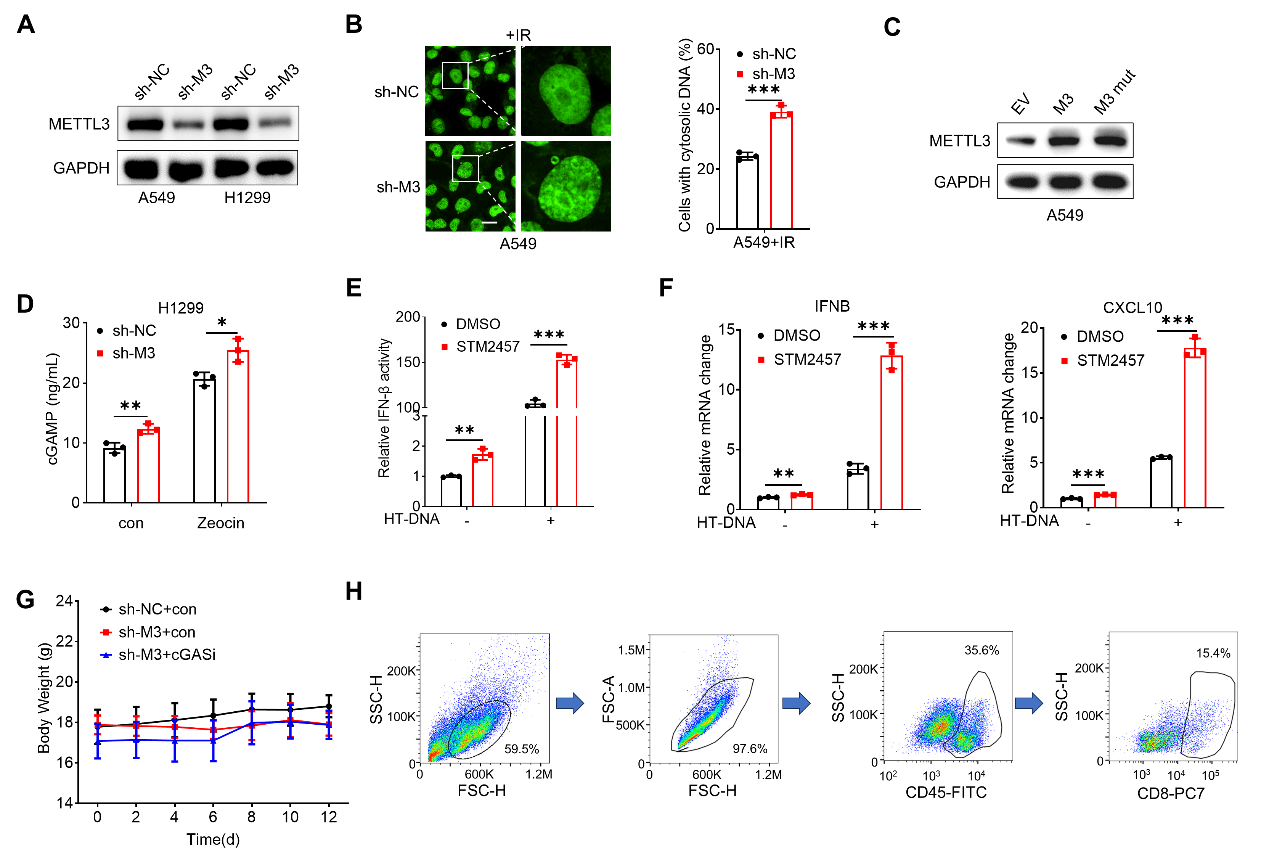
**

**Supplementary Figure 2 Knockdown of METTL3 promotes the innate immune response and activates the cGAS-STING pathway.**

1. The protein expression of METTL3 in sh-*METTL3* A549, sh-*METTL3* H1299 cells and their corresponding control cells;
2. Representative confocal images of PicoGreen stain in sh-control and sh-METTL3 A549 cells with IR treatment (left) and the percentages of cells displaying cytosolic DNA were measured (right), scale bar =20 μm;
3. The protein expression of METTL3 in A549 cells transfected with vector control, METTL3 WT plasmid, METTL3 DA mutant plasmid for 24 h;
4. Intracellular cGAMP levels in sh-control and sh-METTL3 H1299 cells with and without Zeocin treatment;
5. The mRNA levels of IFNB1, CXCL0 mRNA in A549 cells pretreated with STM2457 for 24 h and then treated with HT-DNA for 4 h;
6. The IFNB1 promoter activities in sh-control and sh-METTL3 A549 cells treated with HT-DNA;
7. The body weight of mice with sh-control, sh-*METTL3* LLC syngeneic tumor with or without G140 treatment;
8. The animal-associated flow cytometry gating strategies.

Data are presented as mean ± SD from three independent experiments. **p*<0.05, ***p*<0.01, ****p*<0.001, ns, no significance, by Student’s *t* test between two groups and by one-way ANOVA followed by Bonferroni test for multiple comparison.


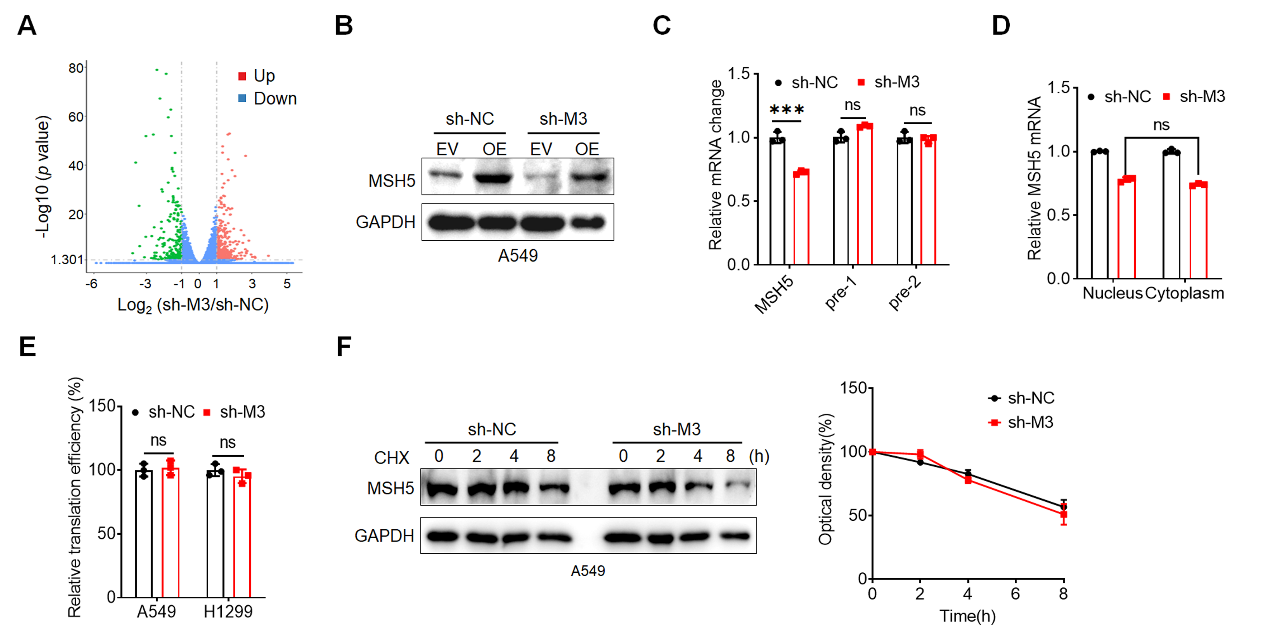


**Supplementary Figure 3 MSH5 mediates METTL3-regulated HR efficacy and cytosolic DNA accumulation.**

1. Volcano plots to determine different genes in sh-*METTL3* cells as compared with that in A549 cells, each dot represents a gene;
2. The protein expression of MSH5 in A549 cells transfected with vector control or MSH5 plasmid for 24 h;
3. The levels of precursor *MSH5* mRNA in sh-control and sh-*METTL3* A549 cells;
4. The relative levels of nuclear versus cytoplasmic *MSH5* mRNA in sh-control and sh-*METTL3* A549 cells;
5. The translation efficiency of endogenous MSH5 in sh-control and sh-*METTL3* A549 cells;
6. Cells were treated with 10 μg/ml CHX for the indicated periods, and the protein expression of MSH5 was detected by western blot analysis (left) and quantitatively analyzed (right).

Data are presented as mean ± SD from three independent experiments. ****p*<0.001, ns, no significance, by Student’s *t* test between two groups and by one-way ANOVA followed by Bonferroni test for multiple comparison.


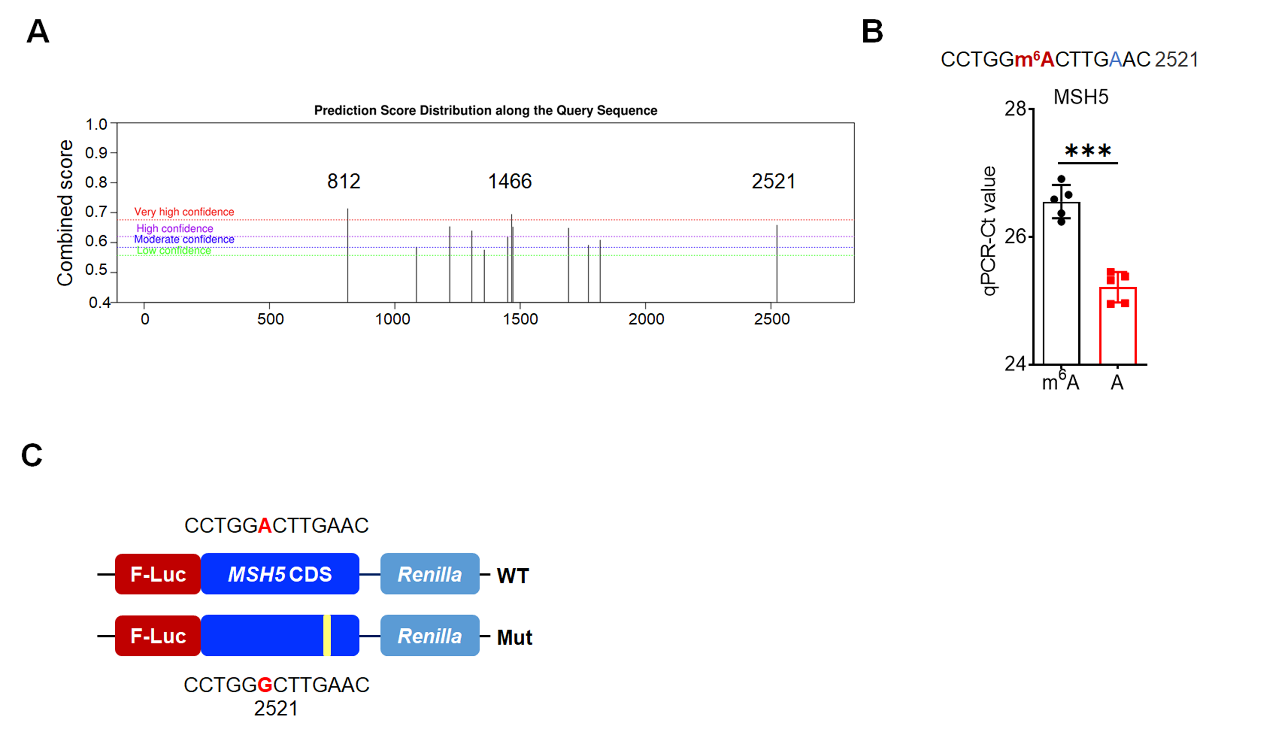


**Supplementary Figure 4 METTL3 stabilized MSH5 mRNA via binding IGF2BP2 with methylation of A2521.**

1. The predicted m^6^A peaks in *MSH5* mRNA from the m^6^A sites predictor SRAMP;
2. The threshold cycle (Ct) of qPCR showing SELECT results for detecting m^6^A site in the potential m^6^A site (A2521) and negative A site (A2526) of MSH5 in A549 cells;
3. Schematic representation of mutation in CDS to investigate the m^6^A roles on MSH5 expression.

Data are presented as mean ± SD from three independent experiments. ****p*<0.001, by Student’s *t* test between two groups and by one-way ANOVA followed by Bonferroni test for multiple comparisons.


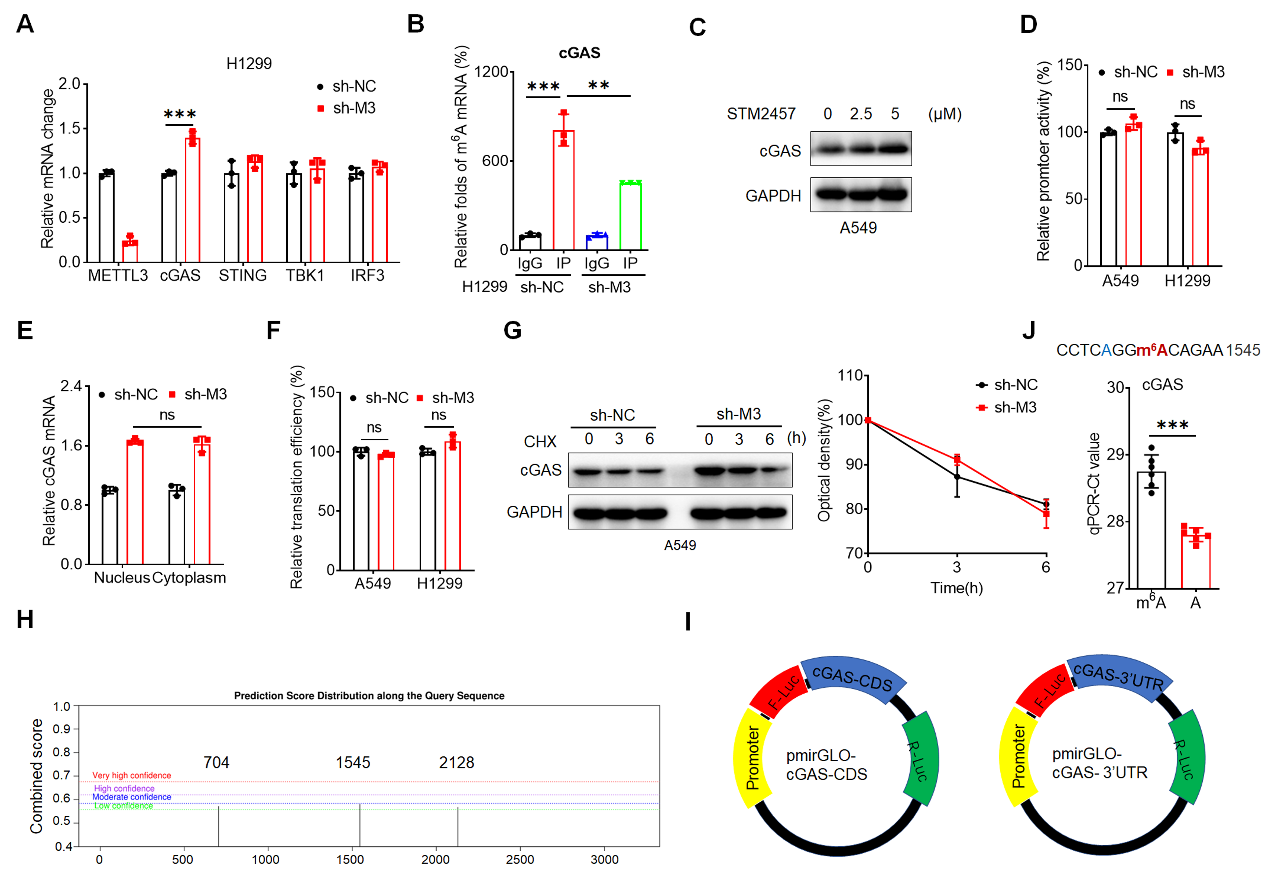


**Supplementary Figure 5 METTL3 destabilized cGAS mRNA via binding YTHDF2 and A1545 methylation**

1. The mRNA expression of cGAS, STING, TBK1, IRF3 in sh-control and sh-METTL3 H1299 cells;
2. m^6^A RIP-qPCR analysis of cGAS in sh-control and sh-*METTL3* H1299 cells;
3. The protein expression of cGAS in A549 cells treated with STM2457;
4. Cells were transfected with pGL3-Basic- cGAS -luc reporter and pRL-TK plasmid for 24 h, the promoter activities were presented as the ratios of the reporter normalized to pRL-TK plasmid;
5. The relative levels of nuclear versus cytoplasmic cGAS mRNA in sh-control and sh-*METTL3* A549 cells;
6. The translation efficiency of endogenous cGAS in sh-control and sh-*METTL3* A549 cells;
7. Cells were treated with 10 μg/ml CHX for the indicated periods, and the protein expression of cGAS was detected by western blot analysis (left) and quantitatively analyzed (right);
8. The predicted m^6^A peaks in *cGAS* mRNA from the m^6^A sites predictor SRAMP;
9. Schematic representation of pmirGLO-*cGAS* CDS and pmirGLO-*cGAS* 3’UTR reporter;
10. The threshold cycle (Ct) of qPCR showing SELECT results for detecting m^6^A site in the potential m^6^A site (A1545) and negative A site (A1542) of cGAS in A549 cells;

Data are presented as mean ± SD from three independent experiments. ***p*<0.01, ****p*<0.001, ns, no significance, by Student’s *t* test between two groups and by one-way ANOVA followed by Bonferroni test for multiple comparison.


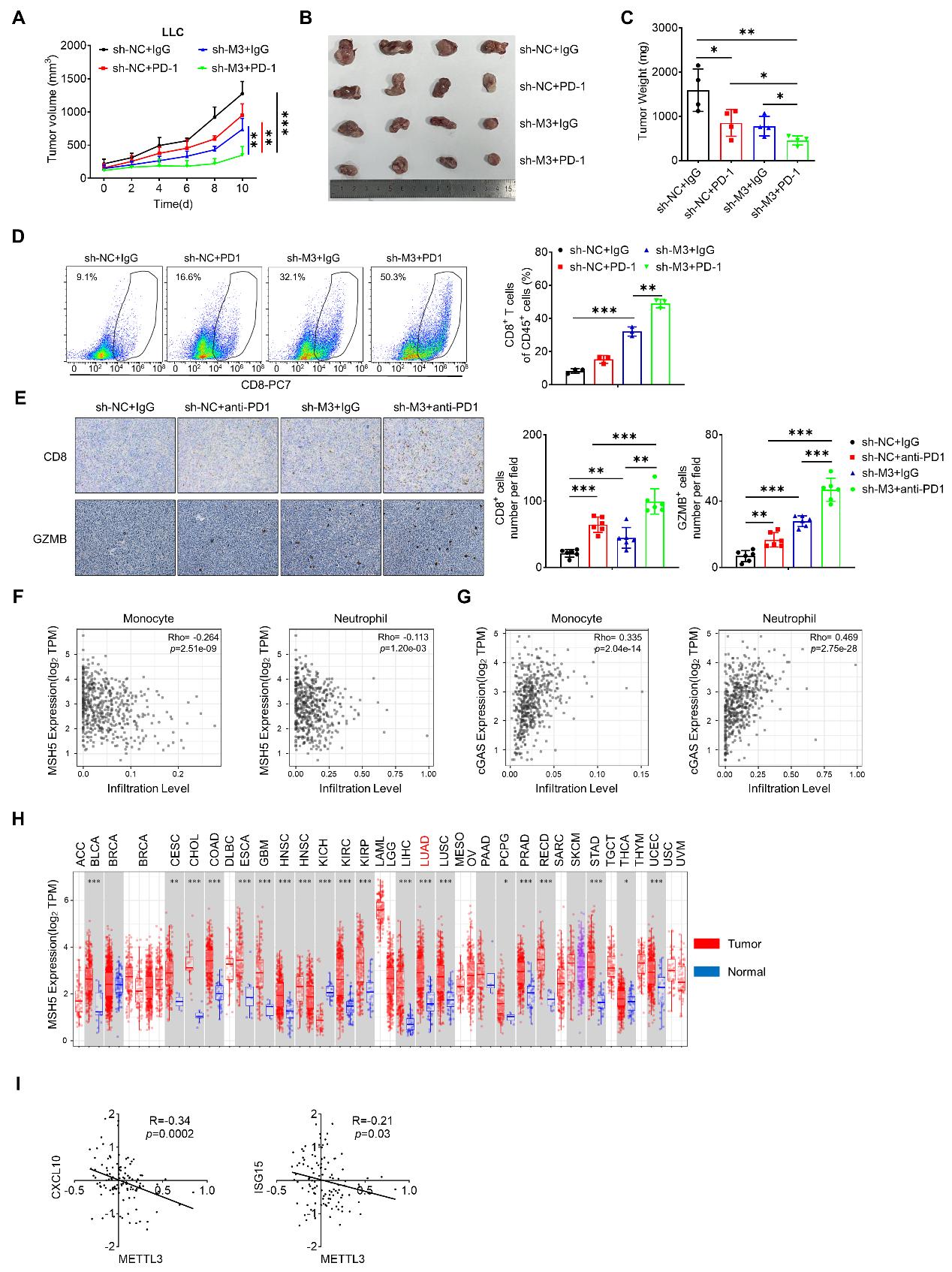


**Supplementary Figure 6 METTL3 inhibition enhances immunotherapy and suppresses cancer progression in LUAD.**

(A~C) The tumor growth curves (A), the tumor volume (B), and the tumor weight (C) of syngeneic tumor models using sh-control or sh-METTL3 LLC cells treated with or without anti-PD-1 antibody;

1. The percentages of CD8^+^ T cells in CD45^+^ cells in the tumor tissues taken from mice with sh-control or sh-METTL3 LLC cells syngeneic tumor treated with or without anti-PD-1 antibody;
2. IHC (CD8 and GZMB)-stained paraffin-embedded sections obtained from sh-control or sh-METTL3 LLC cells syngeneic tumor treated with or without anti-PD-1 antibody, the scale bar is 100 μm.
3. The correlation between the expression of MSH5 and infiltrating level of monocytes, or neutrophils in LUAD based on the TIMER platform;
4. The correlation between the expression of cGAS and infiltrating level of monocytes, or neutrophils in LUAD based on the TIMER platform;
5. The expression of MSH5 across different types of cancers based on the TCGA database;
6. The correlation between the expression of METTL3 and CXCL10, ISG15 in LUAD from CPTAC database;

Data are presented as mean ± SD from three independent experiments. **p*<0.05, ***p*<0.01, ****p*<0.001, by Student’s *t* test between two groups and by one-way ANOVA followed by Bonferroni test for multiple comparisons.

**Supplementary Table legends**

Table S1 Different expression genes in sh-METTL3 A549 cells;

Table S2 HR-relate genes;

Table S3 m^6^A-modified genes in A549 cells;

Table S4 Primers used in the present study.

**Materials and methods**

- 1. **Cell line and cell culture**

Human A549, H1299, and murine LLC, LA795 cells were commercially obtained from American Tissue Cell Culture (ATCC, USA) and maintained by our laboratory. Cells were cultured in high-glucose DMEM (GIBIO, USA) medium supplemented with 10% FBS (GIBIO, USA) and 100 U/ml penicillin/streptomycin (Beyotime, China) under an atmosphere of 5% CO2 at 37℃. The stable cell lines were cultured in a medium containing puromycin or neomycin until 3 days before the experiment.

- 1. **Plasmid, siRNA, shRNA, and generation of stable cell lines**

The cDNA of MSH5 was cloned into the pcDNA3 vector (Invitrogen, USA), and the CDS of METTL3 was cloned into the ppB vector to generate an over-expression plasmid. In contrast, the METTL3 mutant DA (D395A) plasmid was generated in our previous study[1]. The following siRNAs were synthesized (Ribobio, China) and used in the survey: siRNA negative control (si-NC): 5’-UUC UCC GAA CGU GUC ACG U-3’; IGF2BP2 #1: 5’-CAT GCC GCA TGA TTC TTG A-3’; IGF2BP2 #2: 5’-GAA CGA ACT GCA GAA CTT A-3’; IGF2BP2 #3: 5’-AAC AGG GAC CAA GAT AAC A-3’; YTHDF2 #1: 5’-GAC CAA GAA TGG CAT TGC A-3’; YTHDF2 #2: 5’- GCA CAG AAG TTG CAA GCA A-3’. To generate stable cell lines with continuous suppression of METTL3, cells were transfected with lentivirus-shRNA for negative control and METTL3 before selection with puromycin.

- 1. **Western blot analysis**

Cells were washed with PBS, lysed in radio-immunoprecipitation assay (RIPA) buffer containing 1 mM PMSF (Beyotime, China), and placed on ice for 30 min. Then, cells were centrifuged at 12,000×g for 20 min, and the protein concentration was determined using the BCA Protein Assay Kit (Thermo Fisher, USA). Proteins were separated by 10% SDS-PAGE gel and electro-transferred to polyvinylidene difluoride membrane (Bio-Rad, USA). The membranes were blocked with 5% nonfat milk in 1×PBST for 30 min at room temperature, and incubated at 4℃ overnight with the following primary antibodies: anti-METTL3 (15073-1-AP, proteintech, China); anti-YTHDF2 (ab220163, Abcam, England); anti-YTHDF1 (ab99080, Abcam, England); Anti-IGF2BP1 (8482S, CST, USA); Anti-IGF2BP2 (14672S, CST, USA); Anti-IGF2BP3 (25864S, CST, USA); Anti-γH2AX (2577, CST, USA); Anti-cGAS (A25686, Abclonal Technology, China); Anti-STING (A21051, Abclonal Technology, China); Anti-p-STING (AP1369, Abclonal Technology, China). Anti-GAPDH (5174Ss, CST, USA) was used as an internal loading control. After incubation with corresponding secondary antibodies (CST, USA), the membranes were incubated with ECL substrate (Thermo Fisher, USA).

- 1. **m^6^A-RIP qPCR**

We performed m^6^A qPCR using Magna MeRIP™ m^6^A Kit (Millipore, MA) by manufacturer’s protocol. Briefly, 200 μg total RNA was isolated and randomly fragmented with chemical reagents treatment followed by the immunoprecipitation with 5 μg m^6^A antibody or mouse IgG linked to Magna ChIP Protein A/G Magnetic Beads. After extensive washing with IP Buffer, the beads were treated with proteinase K for 30 min at 55℃ with occasional shaking. RNA was purified from the supernatant using TRIzol Reagent following the manufacturer’s instructions. Interested mRNA levels in the elute were measured by RT-qPCR.

- 1. **RIP-RT-PCR**

Two 10-cm plates of cells were washed twice with cold PBS before being collected. 400 μl IP lysis buffer (150 mM KCl, 25 mM Tris (pH 7.4), 5 mM EDTA, 0.5 mM DTT, 0.5% NP40, 1×protease inhibitor, 1 U/μl RNase inhibitor) was added, and resuspended it on ice. The lysate was centrifuged at 12,000×g for 10 min. Then Magnetic beads pre-coated with 4 μl targeted antibodies or mouse IgG (NEB, USA) were incubated with sufficient cell lysates at 4℃ overnight. The beads containing immunoprecipitated RNA-protein complex were treated with proteinase K to remove proteins. Then interested RNAs were purified by TRIzol methods and detected by RT-qPCR with the normalization to input.

- 1. **Sub-cellular fraction**

Fractionation of nuclear and cytoplasmic samples was conducted using Nuclear and Cytoplasmic Extraction Kit (Beyotime, China) according to the manufacturer’s guidelines.

- 1. **Protein stability**

To measure protein stability, cells were seeded in 6-well plates and treated with cycloheximide (CHX, Catalog #14126, Cayman, USA) at a final concentration of 20 μg/ml during indicated times. Cells were collected and lysed in a lysis buffer. Protein expression was measured through western blot analysis.

- 1. **mRNA stability**

To measure RNA stability in tumor cells, actinomycin D (Act-D, Catalog #A9415, Sigma, USA) at 10 μg/ml was added to cells in 6-well plates. After incubation at the indicated times, cells were collected, and RNA was isolated for real-time PCR. The half-life (t_1/2_) of mRNA was calculated using ln2/slope and 18S was used for normalization.

- 1. **SELECT**

SELECT qPCR was conducted by following Xiao’s protocol[2] and our previous study[3]. Briefly, 1500 ng of total RNA was mixed with 40 nM up and down primers and 5 μM dNTP in 17 μl 1×CutSmart buffer (NEB, China). The mixture was incubated with the following program: 90℃ for 1 min, 80℃ for 1 min, 70℃ for 1 min, 60℃ for 1 min, 50℃ for 1 min and 40℃ for 6 min. The sample was further mixed with 0.5 U SplintR ligase, 10 nM ATP, and 3 μl of 0.01 U Bst 2.0 DNA polymerase and incubated at 40℃ for 20 min and denatured at 80℃ for 20 min. Afterward, 20 μl qPCR reaction containing 2 μl of the final reaction mixture, 2×SYBR Green Master Mix (Takara, Japan), and 200 nM SELECT primers were performed. The qPCR program was 95℃, 5 min; (95℃, 10 s; 60℃, 35 s)×40 cycles; 95℃, 15 s; 60℃, 1 min; 95℃, 15 s; 4℃ hold. Results were calculated by normalizing the Ct values of samples to their corresponding Ct values of control. All assays were performed with three independent experiments.

- 1. **Animal experiments**

Animal research received permission from the Animal Experimentation Ethics Committee of Sun Yat-sen University (Nos. 2023002199, 2024000976, 2024001216, and 2025002717) and abided by all relevant ethical regulations. All mice were maintained in the Laboratory Animal Service Center of Sun Yat-Sen University. sh-control, sh-*METTL3* LLC cells (1× 10^6^ per mouse, n = 5 for each group) were diluted in 100 μl of PBS and subcutaneously injected into female C57BL/6J mice to investigate tumor growth. After implantation, the tumor mass was measured with an electronic caliper twice a week, and the tumor volume was calculated according to the following formula: tumor volume (mm^3^) = 0.5 × length × width^2^. When the tumor volume reached about 100 mm^3^, the mice were randomly divided into groups and injected intraperitoneally (ip) at a dose of 30 mg/kg body weight of G140, 100 μg of anti-PD-1 antibody, 50 mg/kg body weight of Olaparib, or a combination of Olaparib and anti-PD-1 antibody treatments every other day. Tumor volume and body weights were recorded every other day after drug treatment and growth curves were plotted using average tumor volume within each experimental group at the set time points. At the end of the observation period, the animals were euthanized by cervical dislocation, and the tumor bulks were peeled off and weighed.

- 1. **Database (DB) Search**

We used the TIMER2.0 web server (http://timer.comp-genomics.org/) to investigate the correlation between METTL3 expression and immune cell infiltration. Correlations between METTL3 protein expression in LUAD tissues and other proteins such as cGAS were extracted from the CPTAC database. The mRNA expression of METTL3 and MSH5 in LUAD and normal tissues was analyzed in the TCGA database.

**Supplementary References**

1. Li ZH, Peng YX, Li JX, Chen ZJ, Chen F, Tu J, Lin SB, Wang HS: **N-6-methyladenosine regulates glycolysis of cancer cells through PDK4.** *Nature Communications* 2020, **11:**2578

2. Xiao Y, Wang Y, Tang Q, Wei L, Zhang X, Jia G: **An Elongation- and Ligation-Based qPCR Amplification Method for the Radiolabeling-Free Detection of Locus-Specific N(6) -Methyladenosine Modification.** *Angew Chem Int Ed Engl* 2018, **57:**15995-16000.

3. Li J, Chen Z, Chen F, Xie G, Ling Y, Peng Y, Lin Y, Luo N, Chiang CM, Wang H: **Targeted mRNA demethylation using an engineered dCas13b-ALKBH5 fusion protein.** *Nucleic Acids Res* 2020, **48:**5684-5694.
